# Optimizing Information in Next-Generation-Sequencing (NGS) Reads for Improving *De Novo* Genome Assembly

**DOI:** 10.1371/journal.pone.0069503

**Published:** 2013-07-29

**Authors:** Tsunglin Liu, Cheng-Hung Tsai, Wen-Bin Lee, Jung-Hsien Chiang

**Affiliations:** 1 Institute of Bioinformatics and Biosignal Transduction, National Cheng Kung University, Tainan, Taiwan; 2 Department of Computer Science and Information Engineering, National Cheng Kung University, Tainan, Taiwan; Radboud University Medical Centre, NCMLS, Netherlands

## Abstract

Next-Generation-Sequencing is advantageous because of its much higher data throughput and much lower cost compared with the traditional Sanger method. However, NGS reads are shorter than Sanger reads, making *de novo* genome assembly very challenging. Because genome assembly is essential for all downstream biological studies, great efforts have been made to enhance the completeness of genome assembly, which requires the presence of long reads or long distance information. To improve *de novo* genome assembly, we develop a computational program, ARF-PE, to increase the length of Illumina reads. ARF-PE takes as input Illumina paired-end (PE) reads and recovers the original DNA fragments from which two ends the paired reads are obtained. On the PE data of four bacteria, ARF-PE recovered >87% of the DNA fragments and achieved >98% of perfect DNA fragment recovery. Using Velvet, SOAPdenovo, Newbler, and CABOG, we evaluated the benefits of recovered DNA fragments to genome assembly. For all four bacteria, the recovered DNA fragments increased the assembly contiguity. For example, the N50 lengths of the *P. brasiliensis* contigs assembled by SOAPdenovo and Newbler increased from 80,524 bp to 166,573 bp and from 80,655 bp to 193,388 bp, respectively. ARF-PE also increased assembly accuracy in many cases. On the PE data of two fungi and a human chromosome, ARF-PE doubled and tripled the N50 length. However, the assembly accuracies dropped, but still remained >91%. In general, ARF-PE can increase both assembly contiguity and accuracy for bacterial genomes. For complex eukaryotic genomes, ARF-PE is promising because it raises assembly contiguity. But future error correction is needed for ARF-PE to also increase the assembly accuracy. ARF-PE is freely available at http://140.116.235.124/~tliu/arf-pe/.

## Introduction

Next-generation-sequencing has transformed recent biological studies [1], including genome assembly. Compared with the traditional Sanger method [2], the throughput of NGS data is much higher and the cost is much lower [3]. Because of these advantages, the number of genome projects has been increasing significantly [4].

NGS, however, poses new computational challenges to *de novo* genome assembly [5], [6]. One big challenge stems from the short length of NGS reads. Reads from all NGS platforms (454 [7]: ∼400 bp, Illumina [8]: 150 bp, ABI SOLiD [8]: 75 bp) are shorter than Sanger reads (800∼1000 bp). Although the new version of 454 machine can generate ∼800 bp reads, only parts of the data reach this length. Short read length is problematic with the presence of repetitive sequences (called repeats) in genomes [6]. When a read comes from a repeat and is shorter than the repeat, it is not clear from which repeat copy the read is obtained. During assembly, mis-joins of genomic regions may occur through the repeat. Repeats exist in almost all genomes, and the problem is more serious for complex eukaryotic genomes [9]. For example, nearly half of the human genome resides in repeats [10]. Because of repeats, it is rare that current assemblers can assemble NGS reads into one complete genome at one shot even for small microbial genomes.

To tackle problems due to short NGS reads, many companies and laboratories are developing new sequencers to increase read length while maintaining or raising data throughput [11]. For example, the single-molecule-real-time sequencer of Pacific Biosciences produces reads of length ∼2000–3000 bp [12], [13]. However, this technology is not yet stable in terms of read quality and the data throughput is still relatively low [3].

Computationally, it is possible to lengthen NGS reads using paired-end (PE) reads. A PE consists of two reads at the two ends of a DNA fragment. When the length of a DNA fragment is shorter than twice the read length, the two reads overlap, which allows them to be merged into one longer read, corresponding to the original DNA fragment. This idea has been implemented in several programs, e.g., SHERA [14], FLASH [15], and COPE [16]. In these studies, the longer merged reads have been shown to improve *de novo* genome assembly. This approach, however, sets a hard limit on length of the recovered DNA fragments, which must be less than twice the read length.

We present a computational tool, ARF-PE, an **A**ssembly-assisted **R**ecoverer of **F**ragments from **P**aired-**E**nd reads. ARF-PE recovers DNA fragments from paired reads that do not overlap. That is, ARF-PE can obtain the unknown sequence in between two paired reads. The upper limit on length of the recovered DNA fragments is thus set by the fragment lengths. Current PE technology can produce PEs from DNA fragments longer than twice the read length. For example, the fragment lengths of Illumina PEs can be ∼500 bp, longer than the twice the read length (e.g., 2*150 = 300 bp). On the overlapping paired reads, ARF-PE outperformed current tools in terms of both quantity and accuracy. On the non-overlapping PE reads of four bacteria and three eukaryotes, the DNA fragments recovered by ARF-PE improved assembly contiguity in almost all cases.

In this work, we demonstrate ARF-PE’s ability to increase Illumina read length from 100 bp to 300∼500 bp, in the range of 454 read lengths. As 454 reads are more expensive than Illumina reads, ARF-PE is economical in obtaining long NGS reads for improving genome assembly. ARF-PE contributes to the field of genome assembly by enhancing assembly completeness, which benefits various downstream biological studies.

## Materials and Methods

### Illumina PE Data and Genomic Sequences

For comparing tools, we obtained the PE libraries simulated at error rates 0, 1, 2, 3, and 5% from the FLASH study [15]. The fragment lengths of these libraries are short (∼180 bp) and the majority of the paired reads (read length 100 bp) overlap.

For testing ARF-PE on PEs of median (∼300 bp) and long (∼500 bp) fragment lengths, we used both simulated and real data. From NCBI Genome and Sequence Read Archive (SRA) databases [17], we downloaded four bacterial genomes (*Cyclobacterium marinum, Escherichia coli, Planctomyces brasiliensis,* and *Spirochaeta smaragdinae*) and their real PE data (Table 1), respectively, for simulation (see below) and analysis.

**Table 1:** Genomic sequences and NGS data used in this study.

Species	NCBI accession of referencegenome (genome size)	SRA accession of Illumina PE library(read and mean fragment length)
*Cyclobacterium marinum* DSM 745	NC_015914 (6,221,273 bp)	SRR407687 (76 bp and 300 bp)
*Escherichia coli* K12 sub-strain MG1655	NC_000913 (4,639,675 bp)	ERR022075 (100 bp and 500 bp)
*Planctomyces brasiliensis* DSM 5305	NC_015174 (6,006,602 bp)	SRR090599 (76 bp and 300 bp)
*Spirochaeta smaragdinae* DSM 11293	NC_014364 (4,653,970 bp)	SRR407537 (76 bp and 300 bp)
*Saccharomyces cerevisiae* S288c	NC_001133∼148 and NC_001224(12,157,105 bp)	SRR452441 (101 bp and 230 bp)
*Neurospora crassa* OR74A	NC_001570 (38,047,924 bp)	SRR018172∼177 and SRR018184∼186(37 bp and 335 bp)
*Homo sapiens* chr22	NT_028395 (35,717,164 bp)	Simulated reads of length 100 bp;mean fragment length 500 bp

Note that the mean fragment lengths of real data are obtained from the NCBI SRA records.

For testing ARF-PE on PEs of eukaryotic genomes, we downloaded the real PEs of two fungi (*Neurospora crassa* and *Saccharomyces cerevisiae*) from NCBI SRA (Table 1). Besides, we simulated PE reads from human chromosome 22 (Table 1) using PIRS [18] (see below). The chromosome sequence was broken into segments at consecutive N’s for simulating PE reads. For each real dataset, we removed reads containing any N and took PEs of 100X coverage, starting from the file head, for analysis.

### PE Read Simulation

Our simulation captured three features of real PE data: (1) non-uniform read coverage across genome, (2) variation of fragment lengths, and (3) position-dependent error rates on reads. To mimic the non-uniform coverage of reads across genome of real data, we mapped reads to the reference genome using SOAP2 [19] (options: -m 100 -x 500 -l 40–v 4 for the three bacterial SRR datasets, and -m 300 -x 700 -l 40–v 4 for the ERR dataset in Table 1) and counted the number of reads starting at each base. The read-start profile set the probability of generating fragments starting at each base. Given a starting position, we determined a fragment length following a normal distribution with a mean at 300 bp or 500 bp and a standard deviation 10% of the mean fragment length. We then extracted paired reads from the two ends of the fragment and introduced errors to the reads. We calculated average quality score at each base position of real sequences, which was converted into an error rate. The position-dependent error rates were multiplied by a factor for controlling the overall error rate at 1%. We randomly introduced substitution errors according to these error rates. In this procedure, we simulated PE reads of length 76 bp for the three bacterial SRR datasets and 100 bp for the ERR dataset to 100X coverage.

We used PIRS [18] to simulate PE reads from human chromosome 22. PIRS also renders the three features mentioned above. PIRS sets non-uniform read coverage according to local GC content. We used the position-dependent error profile (humNew.PE100.matrix.gz), GC-depth profile (humNew.gcdep_100.dat), and insert-deletion error profile (phixv2.InDel.matrix) provided by the PIRS package to simulate PE reads of length 100 bp (with a mean fragment length 500 bp and standard deviation 50 bp) to 100X coverage.

### ARF-PE Workflow

**Figure 1:**
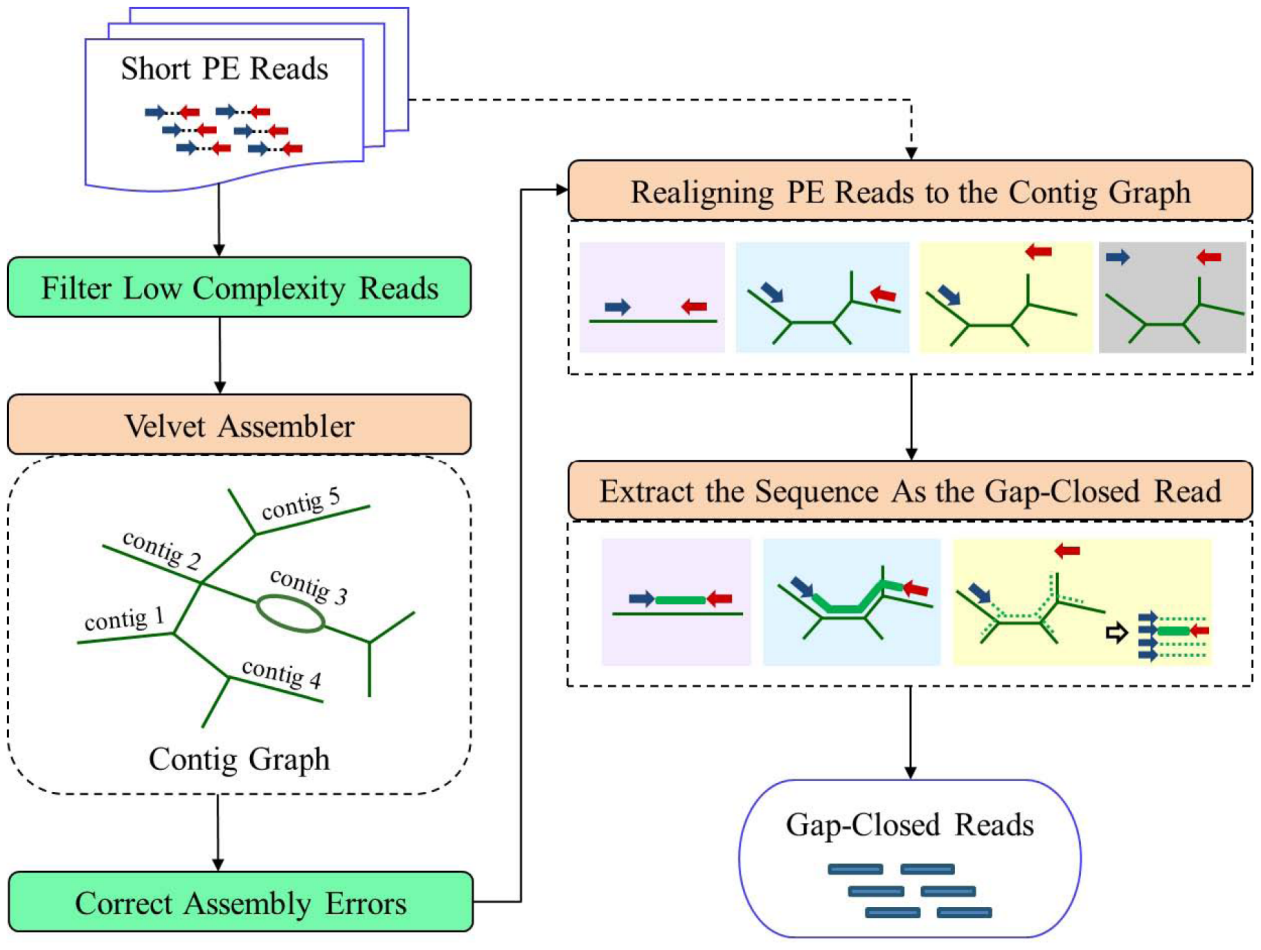
Workflow of ARF-PE. ARF-PE runs in three steps at its kernel (in brown rectangles). First, ARF-PE assembles PE reads into contigs using Velvet and obtains the contig graph. Second, PE reads are re-aligned to the contig sequences. ARF-PE then splits the PEs into four categories based on the mappings of the two reads. Third, ARF-PE extracts the sequences between the two mapped loci as the recovered DNA fragments of PEs. Besides the kernel, ARF-PE offers two options (in green rectangles): filtering low-complexity reads before assembly and correcting errors in contigs after assembly. See main text for details.

ARF-PE runs in three steps (Figure 1). First, it assembles PE reads into contigs using Velvet (v1.2.03) and obtains the contig graph, which describes the connections between contigs. Second, it maps all the PE reads to the assembled contigs using SOAP2 [19]. The PEs are then split into four categories according to the mapped loci. The four categories are (1) regular: both reads mapped on the same contig, (2) bridging: two reads mapped on different contigs, (3) single-mappable-end: only one read mapped, and (4) unmapped: both reads not mapped. Third, ARF-PE searches the contig graph for a path connecting the two mapped loci of each PE. If a path is found, the corresponding sequence is extracted as the recovered DNA fragment. We describe below the searching details for the first three types of PEs.

### a. Regular

For each regular PE, ARF-PE checks whether the two reads are correctly oriented and separated by a proper distance. A regular PE is considered authentic when its two reads are on the opposite strands of the same contig. In addition, the distance between the two mapped loci (i.e., fragment length) should be within a range. The range is set via the distribution of fragment lengths of all regular PEs. We set the minimum and maximum when the frequency of fragment length drops below a cutoff value (five by default). When an authentic regular PE is found, ARF-PE extracts the sequence between the two mapped loci (including the mapped loci) on the contig as the recovered DNA fragment (Figure 1). It is rare that a regular PE is authentic on more than one contig. In such cases, ARF-PE selects the one with the highest mean coverage of reads.

### b. Bridging

For each bridging PE, ARF-PE searches the contig graph for paths of contigs connecting the two mapped contigs with a modified depth-first-search (DFS) algorithm. The modified DFS algorithm considers contig orientations and stops a search when the traversed path exceeds the fragment length maximum. If only one path of contigs is found, the sequence along the corresponding contig path is extracted as the recovered DNA fragment (Figure 1). Otherwise, ARF-PE selects the path with the highest mean coverage of reads.

### c. Single-mappable-end

When only one read of a PE is mapped, the other read may not be mapped because it locates at the junction of two contigs or it contains more errors than allowed. SOAP2 allows at most two mismatches in the seed region of a read; the maximal number of mismatches in the whole read is user-defined. By default, ARF-PE sets the seed length as 40 and maximal number of mismatches as 5. To align the unmapped read of a single-mappable-end, ARF-PE searches the contig graph for all possible sequences extending out from the mapped locus with a similar modified DFS algorithm. It then checks whether the unmapped read can be aligned to these sequences. Specifically, it scans each sequence for a perfect match to the head (10 bases by default) of the unmapped read (Figure 1). Once found, ARF-PE determines the locus of the unmapped read, and extracts the sequence between the two mapped loci as the recovered DNA fragment. Again, if more than one path is found, the path with the highest mean read coverage is selected.

### ARF-PE Options: Read Filtering and Error Correction

ARF-PE offers two options: filtering low complexity reads and correcting assembly errors. By default, a read is considered lowly complex when ≧80% of the bases are identical or when it contains a stretch of 30 identical bases. Low-complexity reads are filtered before Velvet assembly. To correct errors in Velvet assembly, ARF-PE maps PE reads to the assembled contigs using SOAP2. It then collects the reads of authentic regular PEs that are uniquely mapped for building consensus sequences. When a consensus base differs from that on the contig, ARF-PE replaces the contig base with the consensus base if either following criterion is met. First, the base on the contig is not reliable, i.e., in lower-case. Second, the consensus bases constitute at least 60% of the ≧10 covering bases. Note that all the parameter values here are user-adjustable.

### FLASH, and COPE

For comparing tools, we ran FLASH (v1.0.3) and COPE (v1.1.2) on the PE libraries simulated in the FLASH study. For FLASH, we applied default parameters and additionally adjusted the mismatch ratio (option -x) as in the FLASH study. We ran COPE in its full mode (option -m 3), in which read connections were assisted by k-mer frequency. Except setting the quality score offset as 33, we used default parameters.

### *De novo* Genome Assembly

Four assemblers, CABOG (v6.1), Newbler (v2.6), SOAPdenovo (v1.05), and Velvet (v1.2.03), were used to assess the benefits of recovered DNA fragments to *de novo* genome assembly. We used these assemblers to treat three types of data: (1) original PE reads, (2) recovered DNA fragments and the remaining PEs (i.e., whose DNA fragments were not recovered), and (3) recovered DNA fragments and original PEs.

For CABOG, we converted the fastq data and the recovered fragments into fragment format using the command “fastqToCA” and “convert-fasta-to-v2.pl”, respectively, while setting the quality score as 40 for all bases of the recovered DNA fragments. We used the unitigger “bog” and ran the assembly in multi-threads (see below for the full spec file).

doOverlapBasedTrimming = 0; unitigger = bog; bogBreakAtIntersections = 0; bogBadMateDepth = 1000; merylThreads = 8; merOverlapperThreads = 8; merOverlapperExtendConcurrency = 8; merOverlapperSeedConcurrency = 8; ovlThreads = 2; ovlConcurrency = 4; ovlCorrConcurrency = 8; frgCorrThreads = 2; frgCorrConcurrency = 4; merylMemory = 24576; ovlStoreMemory = 24576; doExtendClearRanges = 0; cnsConcurrency = 8.

To run Newbler, we applied the default parameters for assembly. Because Newbler requires quality scores to be in a separate file if there is any, we converted the fastq data accordingly. Recovered DNA fragments were input into Newbler without quality information.

For SOAPdenovo, we scanned possible k-mer values, e.g., from 31 to 81 with a step size 2, and picked the k-mer value resulting in the largest contig N50 length. GapCloser package (v1.12) was applied to further close the gaps between contigs. The results of GapCloser were discussed separately. During assembly, the recovered DNA fragments were used only for building contigs, and all PE data were used for additional scaffolding. We used default parameters of SOAPdenovo except adding an option -d, which removed low-frequency k-mers with single occurrence (see below for a sample configuration file). The maximal read length was set as 400 bp and 600 bp for the PE libraries with an insert size 300 bp and 500 bp. For original PE libraries, the maximal read lengths were set as the read length. For GapCloser, we used default parameters except setting the overlap parameter -p as 21.

max_rd_len = 400.

[LIB].

reverse_seq = 0.

asm_flags = 3.

rank = 1.

avg_ins = 300.

q1 = SRR090599_100X_1.fastq.

q2 = SRR090599_100X_2.fastq.

[LIB].

asm_flags = 1.

f = Merge_All1_new.fa.

We also optimized Velvet assembly via scanning possible k-mer values. By default, Velvet finds the expected coverage and fragment length automatically. Because Velvet outputs scaffolds, we broke the scaffolds into contigs at one or more consecutive N’s. Note that the GAGE script [20] checks only contigs of length at least 200 bp for assembly metrics.

## Results

### Comparison of ARF-PE and Related Tools on Simulated PEs of Short Fragment Lengths

We applied FLASH, COPE, and ARF-PE to the simulated PE libraries of short fragment length (∼180 bp) from the FLASH study (Methods). These data were simulated from the genome of bacterium *Rhodobacter sphaeroides* (size 4.6 Mb). Each library contained 1,000,000 PEs and the read length was 100 bp, accounting for 43.5X read coverage. Note that the majority of the paired reads overlapped. Five simulated PE libraries, with overall error rates 0, 1, 2, 3, and 5%, were used for comparison. In addition, we incorporated the results of SHERA from the FLASH study.

At a zero or low (1 or 2%) error rate, ARF-PE correctly merged more PE reads than SHERA, FLASH, and COPE did (Table 2). As defined in the FLASH study, two paired reads were considered as correctly merged when the length of the merged read equaled that of the corresponding DNA fragment. Note that some bases of a correctly merged read might differ from those of the true DNA fragment. At a zero or low error rate, ARF-PE correctly merged >91% of the PEs while SHERA, FLASH, and COPE correctly merged at most 64%, 70%, and 79% of the PEs, respectively. The number of incorrectly merged PEs by ARF-PE (≦570) was lower than those by SHERA (≧19,044), FLASH (≧1,649), and COPE (≧660). In terms of perfect DNA fragment recovery, ARF-PE was more accurate than FLASH and COPE at low error rates (Table 3). For example, at a 1% error rate, ARF-PE correctly merged 997,136 PEs, among which 993,123 (99.6%) were perfect. In contrast, 17.7% and 55.3% of PEs were perfectly merged by FLASH and COPE, respectively.

**Table 2:** Performance of SHERA, FLASH, COPE, and ARF-PE on merging paired reads.

Error rate	Tool	Correct merge (%)	Incorrect merge (%)	Non-merge (%)
0%	SHERA	637,541 (63.75%)	21,250 (2.13%)	341,199 (34.12%)
	FLASH	700,347 (70.03%)	2,644 (0.26%)	297,009 (29.70%)
	COPE	793,059 (79.31%)	1,208 (0.12%)	205,733 (20.57%)
	ARF-PE	**997,834 (99.78%)**	**388 (0.04%)**	**1,778 (0.18%)**
1%	SHERA	629,166 (62.92%)	20,106 (2.01%)	350,728 (35.07%)
	FLASH	699,177 (69.92%)	2,017 (0.20%)	298,806 (29.88%)
	COPE	678,554 (67.86%)	882 (0.09%)	320,564 (32.06%)
	ARF-PE	**997,136 (99.71%)**	**443 (0.04%)**	**2,421 (0.24%)**
2%	SHERA	617,247 (61.72%)	19,044 (1.9%)	363,709 (36.37%)
	FLASH	686,169 (68.62%)	1,649 (0.16%)	312,182 (31.22%)
	FLASH-0.3	695,797 (69.58%)	4,984 (0.50%)	299,219 (29.92%)
	COPE	618,003 (61.80%)	660 (0.07%)	381,337 (38.13%)
	ARF-PE	919,162 (91.92%)	**570 (0.06%)**	80,268 (8.03%)
	ARF-PE-k49	**994,005 (99.40%)**	2,387 (0.24%)	**3,608 (0.36%)**
3%	SHERA	602,232 (60.22%)	18,138 (1.81%)	379,630 (37.96%)
	FLASH	649,094 (64.91%)	1,393 (0.14%)	349,513 (34.95%)
	FLASH-0.35	690,561 (69.06%)	7,650 (0.77%)	301,789 (30.18%)
	COPE	567,772 (56.78%)	497 (0.05%)	431,731 (43.17%)
	ARF-PE	325,664 (32.57%)	**381 (0.04%)**	673,955 (67.40%)
	ARF-PE-k39	**964,283 (96.43%)**	22,705 (2.27%)	**13,012 (1.30%)**
5%	SHERA	563,706 (56.37%)	17,032 (1.7%)	419,732 (41.97%)
	FLASH	480,145 (48.01%)	1,214 (0.12%)	518,641 (51.86%)
	FLASH-0.35	641,346 (64.13%)	5,733 (0.57%)	352,921 (35.29%)
	COPE	390,206 (39.02%)	282 (0.03%)	609,512 (60.95%)
	ARF-PE	1,505 (0.15%)	**6 (0.00%)**	998,489 (99.85%)
	ARF-PE-k31	**811,263 (81.13%)**	27,548 (2.75%)	**161,189 (16.12%)**

The four tools are applied to merge paired reads simulated from the *R. sphaeroides* genome at various error rates. Following the definition in the FLASH study, a merged read is correct when its length equals the corresponding fragment length. Note that the two categories “correct non-merge” and “incorrect non-merge” in the FLASH study are combined together. Because ARF-PE can merge paired reads with little or no overlap, the two categories do not apply for ARF-PE. For each error rate, the best results are shown in bold.

**Table 3:** Percentage of perfect DNA fragment recoveries by FLASH, COPE, and ARF-PE.

Error rate	Tool	Correct merge (%)	Perfect merge (%)	Non-perfect merge (%)
0%	FLASH	70,0347	700,347 (100.00%)	**0 (0.00%)**
	COPE	793,059	**793,059 (100.00%)**	**0 (0.00%)**
	ARF-PE	**997,834**	994,002 (99.62%)	3,832 (0.38%)
1%	FLASH	699,177	123,907 (17.72%)	575,270 (82.28%)
	COPE	678,554	375,518 (55.34%)	303,036 (44.66%)
	ARF-PE	**997,136**	**993,123 (99.60%)**	**4,013 (0.40%)**
2%	FLASH	686,169	33,648 (4.90%)	652,521 (95.10%)
	FLASH-0.3	695,797	33,684 (4.84%)	662,113 (95.16%)
	COPE	618,003	208,167 (33.68%)	409,836 (66.32%)
	ARF-PE	919,162	914,278 (99.47%)	4,884 (0.53%)
	ARF-PE-k49	**994,005**	**989,208 (99.52%)**	**4,797 (0.48%)**
3%	FLASH	649,094	8,282 (1.28%)	640,812 (98.72%)
	FLASH-0.35	690,561	8,318 (1.20%)	682,243 (98.80%)
	COPE	567,772	127,867 (22.52%)	439,905 (77.48%)
	ARF-PE	325,664	321,808 (98.82%)	**3,856** (1.18%)
	ARF-PE-k39	**964,283**	**957,597 (99.31%)**	6,686 **(0.69%)**
5%	FLASH	480,145	0 (0.00%)	480,145 (100.00%)
	FLASH-0.35	641,346	0 (0.00%)	641,346 (100.00%)
	COPE	390,206	25,530 (6.54%)	364,676 (93.46%)
	ARF-PE	1,505	1,351 (89.77%)	**154** (10.23%)
	ARF-PE-k31	**811,263**	**801,334 (98.78%)**	9,929 **(1.22%)**

On the same data in Table 2, we show the percentage of merged reads that are identical to the corresponding DNA fragments.

At a higher error rate (3 or 5%), the performance of ARF-PE with default parameters dropped significantly (Table 2). This might be explained by the more fragmented Velvet assembly when reads contained more errors. At a 2% error rate, Velvet assembled the PEs into 302 scaffolds, whose N50 length was 38,225 bp. At a 3% error rate, the number of scaffolds increased to 2,761, and the N50 length dropped to 2,651 bp. The low performance could be rescued by tuning the parameter, k-mer length, for Velvet assembly. By default, ARF-PE sets the k-mer length as 60% of read length. At a higher error rate, a smaller k-mer length usually raises assembly contiguity. For example, at a 5% error rate, a k-mer length of 31 raised the scaffold N50 length from 238 bp to 345,382 bp. This enabled ARF-PE to correctly merge 81% of the PEs while SHERA, FLASH, and COPE correctly merged 56%, 64%, and 39% of the PEs, respectively. At a higher error rate, ARF-PE also recovered more perfect DNA fragment than FLASH and COPE did (Table 3).

### Performance of ARF-PE on Simulated PEs of Median and Long Fragment Lengths

We applied ARF-PE to the simulated paired reads that did not overlap. PE libraries of median (∼300 bp) and long (∼500 bp) fragment lengths were simulated from three bacterial and *E. coli* genomes, respectively (Methods). These species were selected because their real PE data were also available. Our simulation captured three features of real PE data: non-uniform coverage of reads across genome, variation in fragment lengths, and position-dependent error rates (Methods). To mimic the real data, we simulated reads of lengths 76 bp and 100 bp for the libraries of median and long fragment lengths, respectively. For each genome, we generated reads of 100X coverage.

Table 4a shows the results of ARF-PE on the simulated PEs of median fragment lengths of *P. brasiliensis*. Of the 3,951,712 PEs, ARF-PE recovered 3,941,755 (99.8%) DNA fragments. Among the recovered DNA fragments, 3,936,750 (99.9%) were correct and 3,912,759 (99.3%) were perfect. Most (97.5%) of the recovered DNA fragments were derived from regular PEs. This was reasonable since most (97.3%) of the PEs were regular. The accuracy of the DNA fragments recovered from regular PEs was higher than from bridging PEs or single-mappable-ends (Table 4a).

**Table 4:** Statistics of DNA fragments recovered from the simulated PEs of bacteria.

(a)					
PE read mappings	Regular	Bridging	Single-mappable-end	Unmapped	Total
No. (%) of PEs	3,844,542 (97.29%)	21,131 (0.53%)	83,820 (2.12%)	2219 (0.06%)	3,951,712 (100%)
No. (%) of recovered fragments	3,844,542 (100.00%)	16,436 (77.78%)	80,777 (96.37%)	N.A.	3,941,755 (99.75%)
No. (%) of correctly recovered fragments	3,842,518 (99.95%)	16,302 (99.18%)	77,930 (96.48%)	N.A.	3,936,750 (99.87%)
No. (%) of perfectly recovered fragments	3,828,589 (99.59%)	11,875 (72.25%)	72,295 (89.50%)	N.A.	3,912,759 (99.26%)
(b)					
No. (%) of PEs	2,214,677 (95.47%)	32,804 (1.41%)	65,033 (2.80%)	7,324 (0.32%)	2,319,838 (100%)
No. (%) of recovered fragments	2,214,677 (100.00%)	28,015 (85.40%)	48,695 (74.88%)	N.A.	2,291,387 (98.77%)
No. (%) of correctly recovered fragments	2,211,883 (99.87%)	27,015 (96.43%)	45,632 (93.71%)	N.A.	2,284,530 (99.70%)
No. (%) of perfectly recovered fragments	2,203,123 (99.48%)	19,295 (68.87%)	39,041 (80.17%)	N.A.	2,261,459 (98.69%)

On the simulated PE libraries of (a) *P. brasiliensis* and (b) *E. coli*, we calculate the percentages of recovered DNA fragments via dividing by the corresponding numbers of PEs. The percentages of correctly and perfectly recovered fragments are calculated via dividing by the number of the recovered DNA fragments for each category of PEs.

ARF-PE performed similarly on the two other simulated PE libraries of median fragment lengths (Table S1). In both cases, ARF-PE recovered >98% of the DNA fragments. Among the recovered DNA fragments, ARF-PE achieved >98% of perfect recoveries. Other features remained similar, e.g., more DNA fragments were recovered from regular PEs than from bridging PEs or single-mappable-ends (Table S1).

The performance of ARF-PE on the PEs of long fragment lengths was similar to that of median fragment lengths. ARF-PE recovered 2,291,387 (98.6%) DNA fragments from the 2,319,838 simulated PEs of the *E. coli* genome (Table 4b). Among those, the percentage of correctly and perfectly recovered DNA fragments were 99.6% and 96.5%, respectively.

### Genome Assembly Including Recovered DNA Fragments: Simulated Data

For each bacteria, we used SOAPdenovo and Newbler to assemble three types of simulated data: (1) original PE reads, (2) recovered DNA fragments and the remaining PEs, and (3) recovered DNA fragments and original PEs (Methods). SOAPdenovo takes a de-Bruijn graph approach for assembly, while Newbler takes an overlap-layout-consensus (OLC) approach. The assembled contigs were then compared to the reference sequences by the GAGE script [20]. Briefly, the GAGE script aligns contigs to the reference genome and detects assembly errors, e.g., SNPs, INDELs, translocation, etc. It breaks contigs at every mis-join and INDEL longer than 5 bp, which were considered as errors in this work. It then outputs several metrics of the original and corrected contigs. Among those, N50 length is a common measure of assembly contiguity, defined so that 50% of the assembled bases are in the contigs of this length or longer. We additionally defined assembly accuracy as the ratio of N50 length of the corrected contigs to that of original contigs.

Table 5 shows the statistics of SOAPdenovo and Newbler assemblies of the *P. brasiliensis* data. To simplify the following texts, claimed changes in assembly metrics were relative to the results of the original PEs unless specified. For SOAPdenovo, the recovered DNA fragments increased the N50 length from 124,055 bp to 193,636 bp and 162,063 bp when being assembled with the remaining and original PEs, respectively. The increment was greater in the Newbler assemblies as the N50 length increased from 45,745 bp to 193,388 bp and 165,897 bp on the second and third types of data, respectively. For all three other bacteria, the recovered DNA fragments also increased the N50 length of both SOAPdenovo and Newbler contigs (Table S2). For all four bacteria except *E. coli*, the increments in N50 length were greater in the Newbler assemblies (2.6∼4.2 fold) than in the SOAPdenovo assemblies (1.3∼1.7 fold).

**Table 5:** Statistics of SOAPdenovo and Newbler assemblies on three types of simulated data of *P. brasiliensis*.

Assembler	Data	Total contiglength (bp)	No. ofcontigs	N50(bp)	No. oferrors	N50 corr.(bp)	Accuracy(%)
SOAPdenovo	original PEs	5,985,166	214	124,055	**0**	124,055	**100.00**
	recovered fragments+remaining PEs	**5,985,261**	**146**	**193,636**	4	156,863	81.01
	recovered fragments+original PEs	6,191,559	204	162,063	**0**	**162,063**	**100.00**
Newbler	original PEs	5,931,886	299	45,745	40	40,444	88.41
	recovered fragments+remaining PEs	**5,956,969**	**102**	**193,388**	**3**	**166,027**	85.85
	recovered fragments+original PEs	5,953,302	104	165,897	4	165,897	**100.00**

Assembly accuracy is defined as the ratio in N50 length after error correction by GAGE. For each assembler and metric, the better results among the three types of data are shown in bold.

In the Newbler assemblies of all four bacteria, the numbers of errors were reduced when the recovered DNA fragments and the original PEs were assembled (Table S2). For *C. marinum*, *P. brasiliensis*, and *S. smaragdinae*, the number of errors dropped from 54, 40, and 52 to 3, 4, and 8, respectively. Consistently, the accuracies of the Newbler assemblies increased respectively from 79.6%, 88.4%, and 71.3% to 100% for the three bacteria. For SOAPdenovo, the number of errors increased in all cases except in the *P. brasiliensis* assemblies of the third type of data. Despite the larger numbers of errors, the assembly accuracy remained the same for *P. brasiliensis*, and *S. smaragdinae*, and dropped from 100% to 96.3% for *C. marinum*. This suggests that the errors occur on shorter contigs. For *E. coli*, the assembly accuracy dropped from 100% to 66.6%. Applying the error correction option of ARF-PE partly rescued the accuracy drop (from 66.6% to 74.7%, data not shown).

We further closed the gaps between SOAPdenovo contigs using GapCloser. On data containing original PEs, GapCloser increased the N50 length for all four bacteria (Table S2). For example, on the *P. brasiliensis* data, GapCloser increased the N50 lengths from 124,055 bp to 218,604 bp and from 162,063 bp to 231,275 bp on the first and third types of data, respectively. On data containing remaining PEs, the N50 length dropped in three of the four bacterial assemblies after applying GapCloser. This is reasonable because GapCloser uses only PEs to close gaps between contigs and the remaining PEs may be too scarce to improve assembly. Below, we focused only on the assemblies of original PEs alone and with the recovered DNA fragments.

With GapCloser, the benefits of recovered DNA fragments to assembly contiguity were less obvious. That is, the N50 lengths were only slightly increased or even decreased when including recovered DNA fragments (Table S2). However, the assembly accuracies increased or remained comparable for all four bacteria except *E. coli*. When we applied the error correction option of ARF-PE, the accuracy of the *E. coli* assembly on the third type of data increased from 71.5% to 98.8% (data not shown).

### Performance of ARF-PE on Real PE Libraries of Median and Long Fragment Lengths

We applied ARF-PE to the real PE libraries of the same four bacteria, three of which were of median fragments lengths (∼300 bp) and one of long fragment lengths (∼500 bp) (Methods, Table 1). To reduce errors in real data, we asked ARF-PE to filter low-complexity reads and correct assembly errors (Methods). Note that the above analyses on simulated data were not subjected to the two options of ARF-PE. On all four datasets, ARF-PE finished within two hours (Table S3). To assess the accuracy of the recovered DNA fragments, we aligned them to the reference genome using BLAT [21]. For each recovered fragment, we took the best genomic segment, i.e., with the largest number of matching bases, as the true DNA fragment.

In general, the percentages of DNA fragments recovered from the real PE data were lower than those from the simulated data, but were still high. For *P. brasiliensis*, the percentage dropped from 99.7% (Table 4a) using simulated data to 87.3% using real data (Table 6a). For all four datasets, ARF-PE recovered >87% of the DNA fragments (Table S4). The overall accuracy of the recovered DNA fragments increased when treating real PE data. For example, the percentage of perfectly recovered fragments slightly increased from 98.7% using simulated data to 98.9% using real data for *E. coli* (Table 6b). In all cases, we observed a higher accuracy of recovered DNA fragments in all three categories of PEs (Table S4). These suggested that the two error correction options enhanced the accuracy of DNA fragment recoveries. Without error correction, the percentage of perfectly recovered DNA fragments indeed dropped in the majority of cases (data not shown).

**Table 6:** Statistics of DNA fragments recovered from the real PEs of bacteria.

(a)					
PE read mappings	Regular	Bridging	Single-mappable-end	Unmapped	Total
No. (%) of PEs	3,319,754 (84.13%)	178,443 (4.52%)	245,865 (6.23%)	201,715 (5.11%)	3,945,777 (100%)
No. (%) of recovered fragments	3,319,754 (100.00%)	9,969 (5.59%)	113,627 (46.22%)	N.A.	3,443,350 (87.27%)
No. (%) of correctly recovered fragments	3,318,586 (99.96%)	9,949 (99.80%)	113,010 (99.46%)	N.A.	3,441,545 (99.95%)
No. (%) of perfectly recovered fragments	3,317,156 (99.92%)	8,207 (82.33%)	111,167 (97.84%)	N.A.	3,436,530 (99.80%)
(b)					
No. (%) of PEs	2,181,071 (94.04%)	37,679 (1.62%)	81,922 (3.53%)	18,656 (0.80%)	2,319,328 (100%)
No. (%) of recovered fragments	2,181,071 (100.00%)	28,368 (75.29%)	43,147 (52.67%)	N.A.	2,252,586 (97.12%)
No. (%) of correctly recovered fragments	2,176,742 (99.80%)	21,237 (74.86%)	38,351 (88.88%)	N.A.	2,236,330 (99.28%)
No. (%) of perfectly recovered fragments	2,173,678 (99.66%)	16,809 (59.25%)	36,223 (83.95%)	N.A.	2,226,710 (98.85%)

On the real PE libraries of (a) *P. brasiliensis* and (b) *E. coli*, we obtain the statistics of the recovered DNA fragments in the same definitions as in Table 4.

### Genome Assembly Including Recovered DNA Fragments: Real Data

We repeated the above assembly and analysis procedures for real data. Besides, we included two more assemblers, Velvet and CABOG, which are another de-Bruijn graph and OLC assembler, respectively. When inferring the benefits of recovered DNA fragments to assembly, we took the better results of the second and third types of data because both contained recovered DNA fragments.

Table 7 shows the assembly statistics of *P. brasiliensis* by four assemblers. For all four assemblers, including the recovered DNA fragments increased assembly contiguity, i.e., the N50 length. The increment was greater for Newbler as the N50 length increased from 80,655 bp to 193,388 bp (2.4 fold) than for other assemblers (1.1∼2.1 fold). Including the recovered DNA fragments also reduced the number of errors from 19 to 6 in the Newbler assemblies. Consistently, the accuracy of Newbler assembly increased from 79.5% to 93.3%. For CABOG, although the number of errors decreased from 23 to 13, the assembly accuracy dropped slightly from 68.6% to 63.5%. For SOAPdenovo, the number of errors increased from one to five after including the recovered DNA fragments, leading to a lower (84.5% v.s. 95.2%) assembly accuracy. The number of errors in the Velvet assemblies increased slightly, but the assembly accuracy increased.

**Table 7:** Statistics of *P. brasiliensis* assemblies by four assemblers on three types of real data.

Assembler	Data	Total contiglength (bp)	No. ofcontigs	N50(bp)	No. oferrors	N50 corr.(bp)	Accuracy(%)
Velvet	original PEs	**5,966,809**	108	158,581	**6**	121,638	76.70
	recovered fragments+remaining PEs	5,965,863	131	**189,520**	10	156,596	82.63
	recovered fragments+original PEs	5,966,059	**97**	178,598	8	**166,495**	**93.22**
SOAPdenovo	original PEs	**5,989,326**	232	80,524	1	76,637	**95.17**
	recovered fragments+remaining PEs	5,982,458	**135**	**166,573**	5	**140,796**	84.53
	recovered fragments+original PEs	5,982,458	**135**	**166,573**	5	**140,796**	84.53
Newbler	original PEs	5,941,846	189	80,655	19	64,086	79.46
	recovered fragments+remaining PEs	**5,957,736**	**93**	**193,388**	**6**	156,799	81.08
	recovered fragments+original PEs	5,955,141	96	**193,388**	**6**	**190,871**	**98.70**
CABOG	original PEs	5,958,614	71	204,964	23	140,673	**68.63**
	recovered fragments+remaining PEs	5,975,578	49	280,361	**13**	177,912	63.46
	recovered fragments+original PEs	**5,979,373**	**45**	**281,920**	15	**178,146**	63.19

For all three other bacteria, most of the above observations held true. For example, the N50 lengths increased after including the recovered DNA fragments in all four bacterial assemblies by all four assemblers except the *S. smaragdinae* assembly by Velvet (Table S5). For all four bacteria, Newbler achieved a greater improvement in N50 length (1.5∼2.7 fold) than SOAPdenovo (1.3∼2.1 fold) and Velvet (∼1.1 fold). The increments in N50 length of the CABOG assemblies (1.4∼4.1 fold) fluctuated more. For all four bacteria, including recovered DNA fragments decreased the number of errors in the Newbler assemblies (Table S5). Consistently, the assembly accuracy increased in all cases using Newbler. For CABOG, the number of errors decreased for all four bacteria, but the assembly accuracy dropped slightly except for *E. coli*, where the accuracy increased from 14.6% to 90.2%. For SOAPdenovo, the number of errors increased in most cases, leading to lower assembly accuracies. The number of errors also increased in most Velvet assemblies, but the assembly accuracy increased for three of the four bacteria. This again suggests that the errors occurred on shorter contigs.

**Figure 2:**
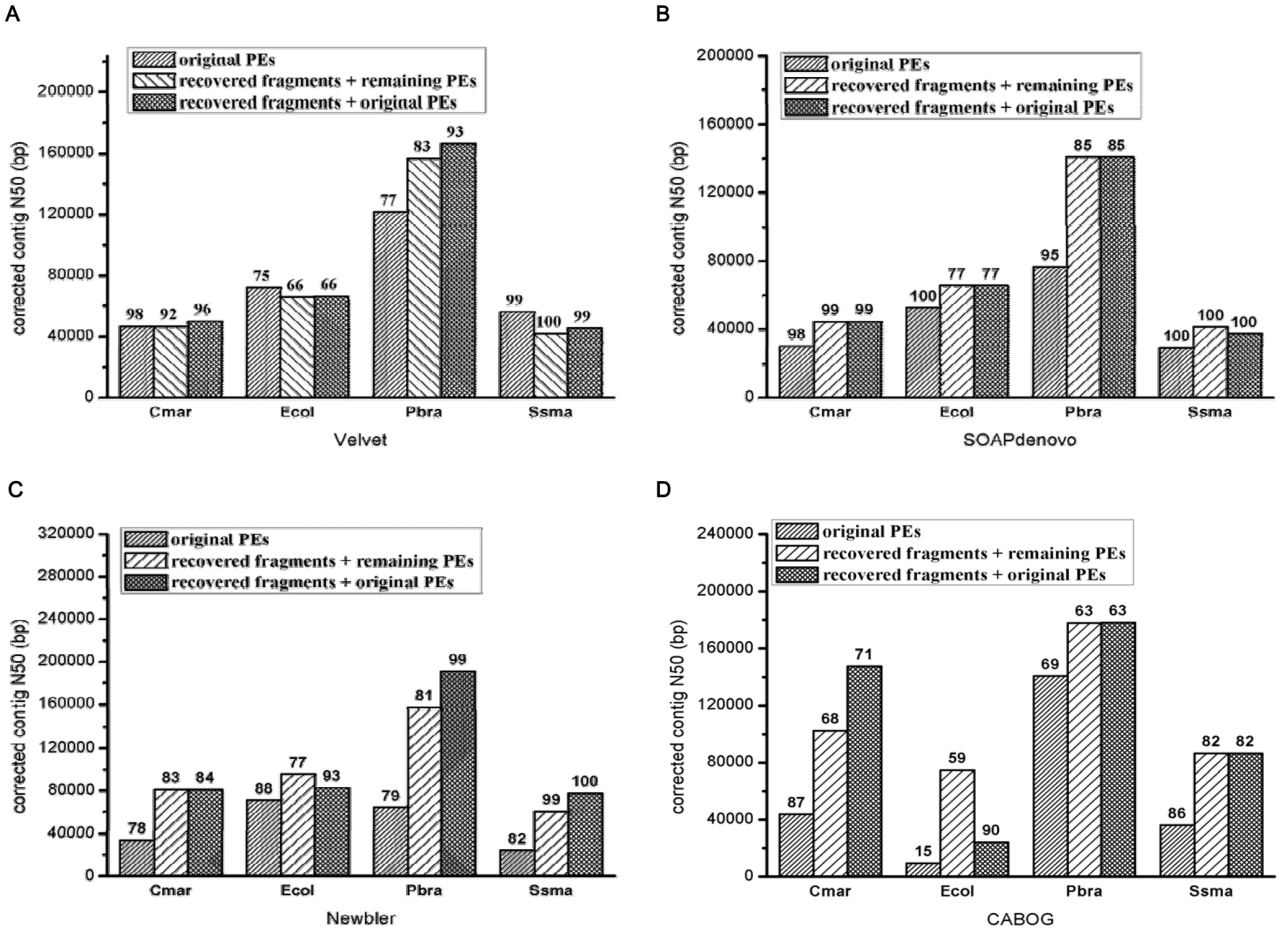
Metrics of four bacterial assemblies by four assemblers. We show the corrected N50 lengths (y-axis) and accuracies (numbers on top of bars) of the four bacterial assemblies by four assemblers: (a) Velvet, (b) SOAPdenovo, (c) Newbler, and (d) CABOG. The accuracy of an assembly is defined as the ratio of the corrected N50 length to the N50 length before correcting assembly errors, and ranges from 0 to 100%. The four species are *C. marinum*, *E. coli*, *P. brasiliensis*, and *S. smaragdinae*. For each species, each assembler treats three types of data: original PE reads, recovered DNA fragments and the remaining PEs, and recovered DNA fragments and original PEs.

We summarized these results using corrected N50 length, which took into account both assembly contiguity and accuracy. Figure 2 shows the corrected N50 length of assembling the three types of data of all four bacteria. The corrected N50 length increased in fourteen of the sixteen cases (four species assemblies by four assemblers) when recovered DNA fragments were included. The improvements by the two OLC assemblers were greater than by the two de-Bruijn graph assemblers.

Similar to the results of simulated data, GapCloser increased the N50 length of all SOAPdenovo assemblies (Table S5). Comparing with the assemblies of original PEs, and with the recovered DNA fragments, the recovered DNA fragments increased the N50 length only for *S. smaragdinae*. However, the assembly accuracy increased for all four bacteria except *S. smaragdinae*, in which assembly the accuracy remained as 100%. Taken together, including the recovered DNA fragments increased the corrected N50 length for all four bacteria except *C. marinum*.

### Effects of Parameters on the Performance of ARF-PE

**Figure 3:**
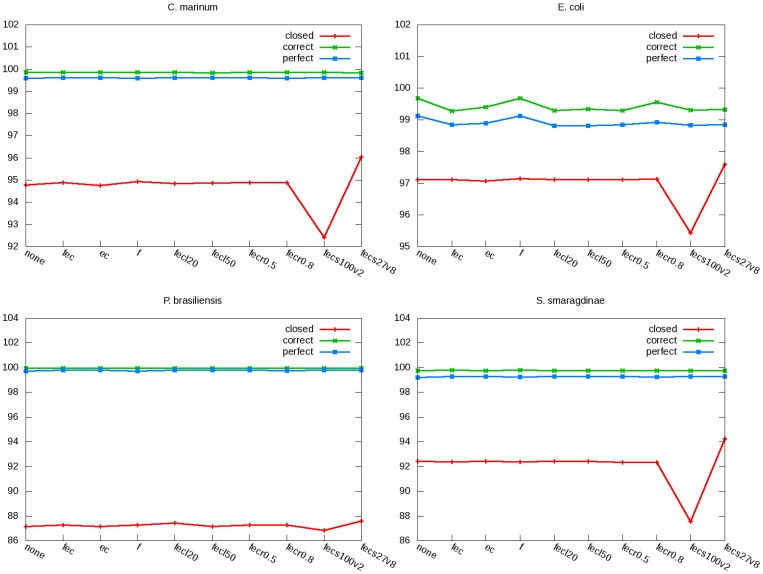
Effects of ARF-PE parameters on the statistics of DNA fragment recovery. Several combinations of ARF-PE parameter values are used to recover DNA fragments from the real data of four bacteria. X-axis indicates the parameter values (f: filtering low-complexity reads, l: minimal number of continuous identical bases for a read to be considered as lowly-complex, ec: error correction to the initial Velvet assembly, r: minimal fraction of consensus bases required for correcting assembly errors, s: seed length of read alignment by SOAP2, v: maximal mismatches allowed in an alignment by SOAP2). Y-axis shows the percentage of DNA fragments that are recovered (red), correctly recovered (green), and perfectly recovered (blue) (the later two values are relative to the number of recovered DNA fragments).

On the real PE data of the four bacteria, we ran ARF-PE with several combinations of parameter values: filtering low-complexity reads or not, minimal number of consecutive identical bases in a low-complexity read (if filtering was applied), correcting errors in the initial Velvet assembly or not, minimal fraction of consensus base for error correction (if applied), and number of mismatches allowed during read alignment by SOAP2. Figure 3 shows the percentage of recovered DNA fragments, and among which the percentages of correctly and perfectly recovered fragments.

For all four bacteria, the percentages of recovered DNA fragments were affected the most by the allowed number of mismatches during read alignment. When fewer mismatches were allowed, more regular PEs became either single-mappable-ends or unmapped PEs (data not shown), which explained the lower percentages of recovered DNA fragments. The effects of the allowed number of mismatches were stronger for *C. marinum*, *E. coli*, and *S. smaragdinae* than for *P. brasiliensis*. For the three bacteria, all other parameters made relatively small changes in the percentage of recovered DNA fragments. For all four bacteria except *E. coli*, the percentages of correct and perfect DNA fragment recoveries remained relatively constant throughout all the explored combinations of parameters. For *E. coli*, the percentages were higher when no error correction was applied, indicating false error corrections.

**Figure 4:**
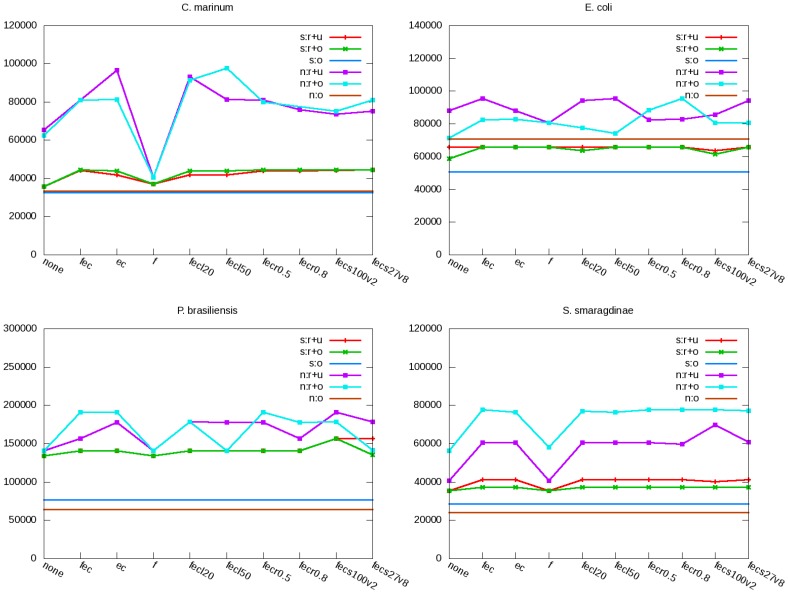
Effects of ARF-PE parameters on genome assembly. SOAPdenovo (s) and Newbler (n) are used to assemble the recovered DNA fragments (r) together with the remaining (u) and original (o) PEs of four bacteria. X-axis indicates the combinations of parameter values defined in Figure 3. Y-axis shows the corrected N50 length when three types of data are assembled.

For each parameter combination, we assembled the recovered DNA fragments with the remaining and original PEs respectively. Figure 4 shows the corrected N50 lengths of the SOAPdenovo and Newbler assemblies. For all four bacteria, SOAPdenovo assemblies were less affected by parameters than Newbler assemblies. The effects also depended on whether the remaining or original PEs were assembled. The differences between data types were greater in the Newbler assemblies than in the SOAPdenovo assemblies. For all four bacteria except *E. coli*, the assemblies without error correction resulted in the smallest corrected N50 length. This held for both assemblers on both data types. The effects of all other parameters were species-, assembler-, and data type-dependent; no consistent trend could be observed. For all explored parameters, the recovered DNA fragments increased the corrected N50 lengths of the SOAPdenovo and Newbler assemblies.

### ARF-PE on the Real Data of Two Fungi

We obtained real Illumina PE libraries of *S. cerevisiae* and *N. crassa* from NCBI SRA (Methods), whose fragment lengths of were about 230 bp and 335 bp, respectively (Table 1). Again, we asked ARF-PE to filter low-complexity reads and correct errors in the initial Velvet assembly. ARF-PE recovered 1,668,173 (75.4%) and 24,973,506 (49.0%) DNA fragments from the 2,212,208 and 50,961,378 PEs of the two fungi, respectively (Table 8). Compared with the results of bacteria, larger fractions of PEs were unmapped (16.4% and 26.0% v.s. <5.2%). Single-mappable-ends constituted 9.0% and 31.4% of the PEs of the two fungi, among which the majority (61.9% and 78.7%, respectively) were not recovered. PEs in these two categories explained most of the non-recovered fragments. For regular PEs, the percentages of correctly (99.2% and 96.1%) and perfectly (99.0% and 95.8%) recovered DNA fragments of the two fungi were slightly lower than those of the four bacteria (>99.8% and >99.6%, respectively). For *S. cerevisiae* and the four bacteria, the percentage of correct and perfect fragments recovered from bridging PEs was comparable (94.2% v.s. >95.4% and 80.3% v.s. 58.6∼88.2%). For *N. crassa*, both the percentages (27.3% and 13.2%) were significantly lower than those of bacteria.

**Table 8:** Statistics of DNA fragments recovered from the PEs of two fungus genomes.

(a)					
PE read mappings	Regular	Bridging	Single-mappable-end	Unmapped	Total
No. (%) of PEs	1,576,567 (71.27%)	73,815 (3.34%)	198,245 (8.96%)	363,581 (16.44%)	2,212,208 (100%)
No. (%) of recovered fragments	1,576,567 (100.00%)	16,047 (21.74%)	75,559 (38.11%)	N.A.	1,668,173 (75.41%)
No. (%) of correctly recovered fragments	1,564,628 (99.24%)	15,110 (94.16%)	70,697 (93.57%)	N.A.	1,650,435 (98.94%)
No. (%) of perfectly recovered fragments	1,561,066 (99.02%)	12,886 (80.30%)	64,941 (85.95%)	N.A.	1,638,893 (98.24%)
(b)					
No. (%) of PEs	21,441,937 (42.07%)	257,884 (0.51%)	16,012,678 (31.42%)	13,248,879 (26.00%)	50,961,378 (100%)
No. (%) of recovered fragments	21,441,937 (100.00%)	116,554 (45.20%)	3,415,015 (21.33%)	N.A.	24,973,506 (49.00%)
No. (%) of correctly recovered fragments	20,612,163 (96.13%)	31,768 (27.26%)	3,121,911 (91.42%)	N.A.	23,765,842 (95.16%)
No. (%) of perfectly recovered fragments	20,533,023 (95.76%)	15,418 (13.23%)	3,018,306 (88.38%)	N.A.	23,566,747 (94.37%)

The statistics are for (a) *S. cerevisiae* and (b) *N. crassa*.

We used SOAPdenovo and Newbler to assess the benefits of recovered DNA fragments to assembly because their performances were more stable than two other assemblers (Figure 2). For *S. cerevisiae*, the recovered DNA fragments reduced the number of SOAPdenovo contigs from 2,282 to 2,006, and increased the N50 length from 10,871 bp to 12,973 bp (Table 9a). With only two more errors, the assembly accuracy went from 99.9% to 99.5%. For Newbler, we showed only the assemblies involving recovered DNA fragments because Newbler could not finish assembling the original PEs in a month. Newbler further reduced the number of contigs to 1,049 and raised the N50 length to 30,652 bp. The accuracy of the Newbler assembly (96.1%) was slightly lower, and the corrected N50 length almost tripled (29,470 bp v.s. 10,860 bp).

**Table 9:** Statistics of SOAPdenovo and Newbler assemblies on three types of real data of two fungi.

(a)							
Assembler	Data	Total contiglength (bp)	No. ofcontigs	N50(bp)	No. oferrors	N50 corr.(bp)	Accuracy(%)
SOAPdenovo	original PEs	11,660,407	2,282	10,871	**11**	10,860	**99.90**
	recovered fragments+remaining PEs	11,683,977	2,075	12,416	13	12,343	99.41
	recovered fragments+original PEs	**11,725,403**	**2,006**	**12,973**	13	**12,901**	99.45
Newbler	recovered fragments	11,496,825	1,388	16,240	35	15,693	**96.63**
	recovered fragments+remaining PEs	11,696,153	**1,049**	**30,652**	**30**	**29,470**	96.14
	recovered fragments+original PEs	**11,695,170**	1,059	**30,652**	32	**29,470**	96.14
(b)							
SOAPdenovo	original PEs	**37,837,899**	19,879	3,420	**150**	**3,388**	**99.06**
	recovered fragments+remaining PEs	34,462,815	**7,923**	**7,233**	1,095	6,604	91.30
	recovered fragments+original PEs	34,462,815	**7,923**	**7,233**	1,095	6,604	91.30
Newbler	recovered fragments	34,515,973	8,108	7,291	1,125	6,656	91.29

For *N. crassa*, the recovered DNA fragments reduced the number of SOAPdenovo contigs from 19,879 to 7,923, raising the N50 length from 3,420 bp to 7,233 bp (Table 9b). Compared with the results of *S. cerevisiae*, the assembly accuracy dropped more (from 99.1% to 91.3%). Taken together, the corrected N50 length almost doubled (from 3,388 bp to 6,604 bp). Note that the assemblies of the recovered DNA fragment with the remaining and original PEs were the same because the maximal k-mer was 127, a value that skipped all PE reads. We used Newbler to assemble only the recovered DNA fragments because the PE reads were too short (37 bp) to be assembled. The statistics of Newbler assembly on the recovered DNA fragments alone were similar to those of the SOAPdenovo assemblies involving the recovered DNA fragments. For example, in the Newbler and SOAPdenovo assemblies, the numbers of contigs were 8,108 and 7,923, and the corrected N50 lengths were 6,656 bp and 6,604 bp, respectively.

On both data of *S. cerevisiae* and *N. crassa*, GapCloser raised the N50 length, but reduced assembly accuracy (Table S6). The accuracy drop was greater for *N. crassa*. Thus, the overall benefits of the recovered DNA fragments to SOAPdenovo+GapCloser assembly were not clear.

### ARF-PE on the Simulated Data of a Human Chromosome

From human chromosome 22, we used PIRS to simulate a PE library whose read length and mean fragment length were 100 bp and 500 bp, respectively (Method). We optimized ARF-PE on this dataset by setting the k-mer value as 81, and applied only the option that corrected assembly errors. From the 17,858,578 PEs, ARF-PE recovered 17,598,427 (98.5%) DNA fragments (Table 10). Among those, 17,306,319 (98.3%) were correct and 16,633,617 (94.5%) were perfect. Most (96.2%) of the PEs were regular, and the percentages of correct and perfect DNA fragments recovered from regular PEs were 99.0% and 96.2%, respectively. Compared with the simulated data of the bacteria, ARF-PE closed a smaller fraction of bridging PEs (66.0% v.s. >73.2%). In this category, the percentages of correctly recovered DNA fragments (81.6% v.s. >95.1%) and the perfect ones (30.8% v.s. >58.7%) were also smaller. A similar trend could be observed for the single-mappable-ends.

**Table 10:** ARF-PE statistics on three types of simulated data of human chromosome 22.

PE read mappings	Regular	Bridging	Single-mappable-end	Unmapped	Total
No. (%) of PEs	17,173,887 (96.17%)	430,632 (2.41%)	236,972 (1.33%)	17087 (0.10%)	17,858,578 (100%)
No. (%) of recovered fragments	17,173,887 (100.00%)	284,044 (65.96%)	140,596 (59.33%)	N.A.	17,598,527 (98.54%)
No. (%) of correctly recovered fragments	16,998,715 (98.98%)	231,900 (81.64%)	75,704 (53.85%)	N.A.	17,306,319 (98.34%)
No. (%) of perfectly recovered fragments	16,517,966 (96.18%)	87,514 (30.81%)	28,137 (20.01%)	N.A.	16,633,617 (94.52%)

Again, we used SOAPdenovo and Newbler to assess the benefits of recovered DNA fragments to the assembly. The total contig lengths of all assemblies (34.1∼35.7 Mb, Table 1) were close to the chromosome size (35.7 Mb). The recovered DNA fragments reduced the number of SOAPdenovo contigs from 7,635 to 3,685 and more than tripled the N50 length (from 15,534 bp to 50,886 bp) (Table 11). Similar to the fungus assemblies, the recovered DNA fragments introduced more errors and the assembly accuracy dropped (from 99.9% to 91.9%). But the corrected N50 length still more than tripled. When only original PEs were assembled, GapCloser raised the N50 length by ∼34 folds (Table S7). But the number of errors increased from one to 209, and the assembly accuracy dropped from 99.9% to 33.6%. With GapCloser, the recovered DNA fragments slightly increased the N50 length (from 526,386 bp to 5,342,034 bp), slightly reduced the number of errors (from 209 and 205), and slightly increased the assembly accuracy (from 33.6% to 37.0%) and the corrected N50 length (from 176,720 bp to 200,383 bp).

**Table 11:** Assembly statistics on three types of simulated data of human chromosome 22.

Assembler	Data	Total contiglength (bp)	No. ofcontigs	N50(bp)	No. oferrors	N50 corr.(bp)	Accuracy(%)
SOAPdenovo	original PEs	**35,658,342**	7,635	15,534	**1**	15,523	**99.93**
	recovered fragments+remaining PEs	34,660,466	**3,685**	**50,886**	123	**46,785**	91.94
	recovered fragments+original PEs	34,660,466	**3,685**	**50,886**	123	**46,785**	91.94
Newbler	recovered fragments+remaining PEs	34,085,423	1,687	76,792	132	62,597	81.52

The Newbler assemblies involving the original PEs could not be finished in one month. In contrast, assembling the recovered DNA fragments and the remaining PEs finished in one day. Compared with the SOAPdenovo assembly on the same data, Newbler further reduced the number of contigs from 3,685 to 1,643 and increased the N50 length from 50,886 bp to 77,012 bp (Table 11). The assembly accuracy was lower (81.5% v.s. 91.9%), but the corrected N50 length was longer (62,597 bp v.s. 46,785 bp). In addition to assembling all the recovered DNA fragments, we randomly sampled 20% of them three times for assembly by Newbler. The results of all the three Newbler assemblies were similar to those when all the recovered DNA fragments were assembled (Table S7). For example, the number of contigs ranged from 1,634 to 1,643 and the corrected N50 length ranged from 62,602 bp to 65,882 bp.

## Discussion

### Selection of Data Sets

We selected the four bacteria unbiasedly. First, we searched the Genomes Online Database [4] for all isolated and completed bacterial genomes. When starting this work, we found 1,766 bacteria with a complete genome. For each bacterium, we queried NCBI SRA for corresponding Illumina PE data and found 340 bacteria with at least one such data. We retained the PE libraries whose read length was 76 bp and fragment length was ∼300 bp. Note that the read length and fragment length of the rest PE libraries were shorter. From the remaining 25 bacteria, we selected those also containing a 454 dataset because we assumed a higher assembly quality when two types of data were involved. Under these criteria, we were left with four bacterial species: *C. marinum, P. brasiliensis, S. smaragdinae, and Streptomyces violaceusniger* (*S. vio*). The initial Velvet assembly of the *S. vio* PE data was much poorer than those of three other species (data not shown). This is reasonable because the *S. vio* genome is of high GC content (70.9%), resulting in highly biased read coverage across the genome. Thus, we did not include the *S. vio* data in our analyses. To find Illumina PE libraries of longer fragment lengths, we queried NCBI SRA with the keywords “whole genome Illumina paired end 500 bacteria” and found the *E. coli* data.

Among the fungi with a complete genome in NCBI, only *N. crassa* OR74A and *Saccharomyces cerevisiae* S288c have Illumina PE data of the corresponding strain in SRA. Requiring genome and data being of the same strain eliminates uncertainty in evaluating assembly accuracy. For human chromosome 22, we resorted to simulated data because the reference sequence and the Illumina PE data in NCBI SRA were likely from different individuals.

### Assembly Optimization

Optimizing assembly is essential for assessing the real benefits of recovered DNA fragments to genome assembly. Data coverage is crucial for assembly performance. Insufficient data clearly lowers assembly contiguity. On the other hand, too much data may deteriorate assembly because errors accumulate. SOAPdenovo assemblies of various amount of real data revealed that N50 length usually saturated when the amount of data reached 100X coverage (Figure S1). Thus, 100X coverage of data avoids a clear fragmented assembly because of scarce data. Except for the data from the FLASH study (used for tool comparison only), all datasets in this study contain reads equal to 100X coverage.

We further optimized the two de-Bruijn graph assemblers by scanning possible k-mer values. Figure S2 shows the impact of k-mer values on the contiguity of Velvet and SOAPdenovo assemblies. The N50 length could drop by an order of magnitude upon a different k-mer value. For CABOG, we used the spec file optimized in the GAGE study. Note that we optimized assembly to render the largest N50 length of contigs. Although not ideal, this strategy is commonly used because longer contigs are usually preferred [20]. In addition to N50 length, we checked assembly accuracy for a more comprehensive evaluation. The N50 length after error correction is used as the major assembly metric because it considers both assembly contiguity and accuracy.

Figure S2 shows that N50 length drops more quickly as k-mer deviates from the best value when original PEs were assembled than when the recovered DNA fragments were included. This indicates another advantage of ARF-PE in assembly optimization. That is, with recovered DNA fragments, one does not need to scan many k-mer values for optimization, which saves computational time. Of course, this requires spending time on ARF-PE and depends on how long each assembly takes. Note that for all organisms in this study except human, we did not optimize ARF-PE by scanning k-mer values for the initial Velvet assembly. Thus, it is possible to further enhance the benefits of recovered DNA fragments to genome assembly.

To fully use recovered DNA fragments by SOAPdenovo, setting maximal read length is important. Without this option, the benefits of recovered DNA fragments to assembly contiguity decreased. That is, on the data containing recovered DNA fragments, the N50 lengths were smaller without setting the maximal read length in almost all cases (Figure S2). When maximal read length was set, N50 length usually plateaued as k-mer value increases. When recovered DNA fragments were assembled, a k-mer value set as the read length or the maximum, 127, gave near optimal assembly in general.

### Simulated and Real Data

We started with simulated data because the true DNA fragments could be readily obtained. To mimic real data, we simulated reads to yield non-uniform coverage similar to that of the real data. Variation in GC content has been known to bias read coverage [22] at library preparation and amplification steps [23]. This often lowers the completeness of genome assembly [24]. Note that we used a different tool, PIRS, to simulate PEs from the human chromosome because we could not find appropriate data for generating non-uniform read coverage profile. PIRS can generate non-uniform coverage of reads based on GC content. However, even with non-uniform coverage, our simulation did not capture every respect of the real data. For all four bacteria, ARF-PE recovered a smaller percentage of DNA fragments using real data than using simulated data. In the SOAPdenovo and Newbler assemblies of the four bacteria on the three types of data, simulated data resulted in a larger N50 length in 22 of the 24 cases (Table S2, Table S5).

The benefits of recovered DNA fragments to assembly are rather similar for simulated and real data. For both simulated and real data, the recovered DNA fragments increased the corrected N50 lengths of all four bacterial assemblies by SOAPdenovo and Newbler. However, the degrees of improvement were less comparable in the Newbler assemblies. We define the degree of improvement as the ratio in corrected N50 length of assembling recovered DNA fragments and original PEs to that of assembling only original PEs. In the Newbler assemblies, the improvements were greater on simulated data (3.2, 1.4, 4.1, and 4.0) than on real data (2.4, 1.2, 3.0, and 3.2) for all four bacteria (*C. marinum*, *E. coli*, *P. brasiliensis*, and *S. smaragdinae*, respectively) (Table S2, Table S5). Using SOAPdenovo, the degrees of improvements on simulated data (1.4, 1.3, 1.3, and 1.7) and on real data (1.5, 1.3, 1.8, and 1.3) were more comparable for all four bacteria.

### Amount of Information and the Utilization

Including recovered DNA fragments for assembly increases the amount of input data. However, the amount of information is the same because recovered DNA fragments are derived from original PEs without extra information. We recommend assembling recovered DNA fragments with original PEs for de-Bruijn graph assemblers because this type of data results in a longer corrected N50 length than other types of data in many bacterial assemblies. For *N. crassa* and human chromosome 22, the optimized SOAPdenovo assemblies skipped PE reads because the optimized k-mer value, 127, was larger than the read length. Thus, for complex eukaryotic genomes, the recovered DNA fragments alone may be enough to optimize assembly. For OLC assemblers, it is not clear which type of data tend to perform better.

It is reasonable that recovered DNA fragments improve Newbler and CABOG assemblies more than Velvet and SOAPdenovo assemblies in general. Newbler and CABOG are initially designed to treat longer NGS single-end reads like 454 reads. Velvet and SOAPdenovo are initially designed to treat short NGS paired-end reads like Illumina PEs. The two de-Bruijn graph assemblers likely have been developed to use PE information relatively well. However, there is still room for improvement as the recovered DNA fragments improved the de-Bruijn graph assemblies in general.

We also find evidences that the two de-Bruijn graph assemblers use longer recovered DNA fragments for assembly. For *C. marinum*, *E. coli*, and *P. brasiliensis*, the Velvet assemblies were optimized at a longer k-mer value when including recovered DNA fragments (from 41 to 67, from 57 to 99, and from 49 to 79, respectively, Figure S2). For SOAPdenovo, assembly optimization occurred at a longer k-mer value for all four bacteria (from 37 to 61, from 51 to 99, from 53 to 117, and from 41 to 91, respectively) (Figure S2).

GapCloser is developed to close the gaps between contigs using PEs. On original PEs, the accuracies of the SOAPdenovo assemblies decreased after applying GapCloser for all four bacteria except *S. smaragdinae*, where the assembly accuracy remained the same (Table S5). In contrast, with the recovered DNA fragments, GapCloser increased the accuracies of the SOAPdenovo assemblies for all four bacteria except *C. marinum*. Thus, we recommend running GapCloser after assembling the DNA fragments recovered by ARF-PE to improve the SOAPdenovo assemblies of bacteria.

For more complex eukaryotic genomes, GapCloser can reduce assembly accuracy significantly. On the original PEs of the human data, GapCloser lowered the assembly accuracy from 99.9% to 33.6% (Table S7). This is consistent with the general impression of the aggressiveness of GapCloser, especially on complex genomes [20]. With GapCloser, although the recovered DNA fragments raised the assembly accuracy from 33.6% to 37.0%, the accuracy was still low. Thus, for complex eukaryotic genomes, the improvement in assembly contiguity by GapCloser can be questionable whatever type of data is used.

### Performance of ARF-PE

The percentage and accuracy of the DNA fragments recovered from bridging PEs and single-mappable-ends were lower than from regular PEs. This is reasonable because the bridging PEs and single-mappable-ends span across the junctions of contig connections, where the accuracy is often lower. During Velvet assembly, a contig may be extended in two or more different ways, indicating the presence of repeats in the genome or sequencing errors. Such ambiguities stop the contig extension, and connect the contig to two or more other contigs. As a result, the sequences near contig connecting junctions are often less accurate.

In general, ARF-PE works well on bacterial and simple eukaryotic genomes. For more complex eukaryotic genomes, ARF-PE still enhances assembly contiguity, but lowers the assembly accuracy, which may be partly explained by the fraction of non-perfectly recovered DNA fragments. For the four bacteria and *S. cerevisiae,* <2% of the recovered DNA fragments were non-perfect. In contrast, >5% of the recovered fragments were non-perfect for *N. crassa* and human chromosome 22. To validate the argument, we separated the perfectly recovered fragments from the non-perfect ones and randomly selected the non-perfect fragments at different fractions. We then assembled the perfect fragments with the non-perfect ones at various fractions using SOAPdenovo. Indeed, the number of errors increased as the fraction of non-perfect fragments increased (Figure S4). For *N. crassa*, 3% of the non-perfect reads resulted in about 154 errors (Figure S4a), which was close to the 150 errors when the original PEs were assembled (Table 9). This suggests a threshold in accuracy of DNA fragment recovery to maintain assembly accuracy when recovered fragments are involved. Unsurprisingly, a similar relationship between assembly errors and fraction of non-perfect fragments was observed for human chromosome 22 (Figure S4b). Thus, reducing errors in the recovered DNA fragments is important for ARF-PE to also raise assembly accuracy for complex eukaryotic genomes. Reducing errors is possible as some recent tools, e.g., iCORN [25] and SEQuel [26], can been applied to fix errors in the initial Velvet assembly. However, as correcting assembly errors is another major topic, we do not explore in detail how they may enhance the usefulness of ARF-PE in this study.

We also note that all the genomes studied here are haploid genomes. Although human genome is diploid, we took one haploid version of chromosome 22 for study. Thus, although ARF-PE can still be applied to polyploidy genomes, but the benefits are not clear yet.

### Effects of ARF-PE Options and Parameters on Assembly

Figure 3 shows that most ARF-PE parameters do not alter much the percentage and accuracy of the recovered DNA fragments. However, assembly accuracy could vary upon various parameter values (Figure 4). This indicates that small differences in recovered DNA fragments can be vital for assembly, which is reasonable because when assembly is done to a certain degree, further improvements require only the reads at the junctions between contigs.

Correcting errors in initial Velvet assembly is crucial as it increased the corrected N50 length in most bacterial assemblies (Figure 4). Read filtering also affects several assemblies, but the effects depend on species, assembler, and data type. Note that for *E. coli*, the two ARF-PE options lowered the accuracy of DNA fragment recovery (Figure 3), but the corrected N50 length increased. We found that the recovered DNA fragments indeed lowered the assembly accuracy, but they increased the N50 length even greater, resulting in a larger N50 length. For example, applying error correction and read filtering lowered the accuracy of the Newbler assembly of recovered DNA fragments and the remaining PEs from 92.9% to 77.0%, but the N50 length increased from 94,926 bp to 123,795 bp (data not shown). As a result, the corrected N50 length increased from 88,221 bp to 95,347 bp.

We further compared the bacterial assemblies with and without error correction and read filtering on the real data of bacteria. Figure S3 shows the differences in number of assembly errors and the ratio in corrected N50 length after the two ARF-PE options. For all four assemblies of the four bacteria, the number of errors decreased in 26 of the 39 cases (Figure S3a, no difference in two cases). Consistent with the above observations for *E. coli*, the number of errors increased after including recovered DNA fragments in all SOAPdenovo and Newbler assemblies (data not shown). The corrected N50 length increased in 34 of the 39 cases (Figure S3b).

The corrected N50 length shown in the main text can be further improved by tuning parameters in general. For example, when error correction was applied without read filtering, the corrected N50 length of the Newbler assembly on the recovered DNA fragments and the remaining PEs further increased for *C. marinum* and *P. brasiliensis*. We also emphasize that for all the explored combinations of parameter values, the recovered DNA fragments increased the corrected N50 length. Thus, parameter tuning should not abolish the benefits of recovered DNA fragments to genome assembly, and only leads to a different degree of improvement.

### Conclusions

In this work, we present a computational tool ARF-PE that increases the lengths of PE reads. ARF-PE takes Illumina PEs as input and recovers the DNA fragments from which two ends the PEs are obtained. On the real data of four bacteria, ARF-PE recovered >87% of the DNA fragments and achieved a >98% accuracy of fragment recovery. The recovered DNA fragments increased the contiguity of all four bacterial assemblies by four popular assemblers. They also increased the accuracies of most bacterial assemblies. On the data of two fungi and human chromosome 22, ARF-PE doubled and tripled the assembly N50 length, respectively, but resulted in slightly lower assembly accuracies. Because long NGS reads like 454 reads are more expensive than Illumina reads, ARF-PE is an economical tool for optimizing Illumina PEs for assembly. Our tool can be run independently or the idea can be incorporated into current assemblers to improve genome assembly. ARF-PE is freely available at http://140.116.235.124/~tliu/arf-pe/under the open source license BSD.

## Supporting Information

Figure S1 **Relationship between contig N50 length and data coverage.** The results are for the SOAPdenovo assemblies (with a k-mer value 51) on the original PE data of *P. brasiliensis*.
(TIFF)

Figure S2 **Relationship between contig N50 length and k-mer value.** For (a) Velvet and (b) SOAPdenovo, we scan various k-mer values to optimize four bacterial assemblies. The black and red curves represent the assembly of original PEs and with recovered DNA fragments, respectively. For SOAPdenovo, the solid and dashed lines are for the assemblies where the parameter maximal read lengths are set and not, respectively.
(TIFF)

Figure S3 **Effects of error correction and read filtering on assembly.** (a) Histogram of differences in number of errors after error correction and read filtering by ARF-PE on the real data of the four bacteria. A negative number stands for a reduction in number of errors. (b) Histogram of the ratio in corrected N50 length. A ratio greater than one means an increase in corrected N50 length after applying the two options of ARF-PE. Note that on real data, the CABOG assembly of recovered DNA fragments and the original PEs of *E. coli* aborted without a clear reason, so there are 39 cases in total.
(TIF)

Figure S4 **Assembly errors by various fractions of non-perfectly recovered DNA fragments.** For (a) *N. crassa* and (b) human chromosome 22, we separate the perfectly recovered DNA fragments from the non-perfect ones. Different fractions of non-perfect fragments (x-axis) and all the perfect ones are assembled by SOAPdenovo (with kmer = 127), and the number of errors (y-axis) are computed by the GAGE script. For each fraction, reads are randomly selected three times for assembly; the mean and variation in number of errors are shown. Horizontal red lines indicate the number of errors when the original PEs are assembled.
(TIF)

Table S1 **Performance of ARF-PE on the simulated PE libraries of four bacteria.** The four species are (a) *C. marinum*, (b) *E. coli*, (c), *P. brasiliensis*, and (d) *S. smaragdinae*. The definitions of percentages follow those in Table 4.
(DOCX)

Table S2 **Assembly statistics on the simulated data of four bacteria by three assemblers.** We use SOAPdenovo, SOAPdenovo+GapCloser, and Newbler to assemble three types of simulated data of four bacteria: (a) *C. marinum*, (b) *E. coli*, (c), *P. brasiliensis*, and (d) *S. smaragdinae*. The three types of data are (1) original PE reads, (2) recovered DNA fragments and the remaining PEs, and (3) recovered DNA fragments and original PE reads.
(DOCX)

Table S3 **ARF-PE runtime on the PE libraries in this study.** The run times are in wall clock seconds. The data of the four bacteria and *S. cerevisiae* are run on a server with four Quad-Core Intel Xeon CPUs E5520 (2.26 GHz) and one with two Quad-Core AMD Opteron processors 2378 (800 MHz), respectively. The *N. crassa* and human data are run on a server with eight Oct-Core Intel Xeon CPUs E7-4820 (2.00 GHz). Note that for each dataset, the total amount of time is not equal to the sum of the section times because ARF-PE spends extra time for data transformation and re-collection.
(DOCX)

Table S4 **Performance of ARF-PE on the real PE libraries of four bacteria.** The four species are (a) *C. marinum*, (b) *E. coli*, (c), *P. brasiliensis*, and (d) *S. smaragdinae*.
(DOCX)

Table S5 **Assembly statistics on the real data of four bacteria.** We use Velvet, SOAPdenovo, SOAPdenovo+GapCloser, Newbler, and CABOG for assembling three types of real data of four bacteria: (a) *C. marinum*, (b) *E. coli*, (c), *P. brasiliensis*, and (d) *S. smaragdinae*.
(DOCX)

Table S6 **Assembly statistics on the real data of two fungi.** The two fungi are (a) *S. cerevisiae*, and (b) *N. crassa*.
(DOCX)

Table S7 **Assembly statistics on the simulated data of human chromosome 22.**
(DOCX)
