# Supplementary figures and images for: Optimizing Information in Next-Generation-Sequencing (NGS) Reads for Improving De Novo Genome Assembly
Source: PLoS One. 2013 Jul 29;8(7):e69503. doi: 10.1371/journal.pone.0069503 (PMC3726674; doi:10.1371/journal.pone.0069503)

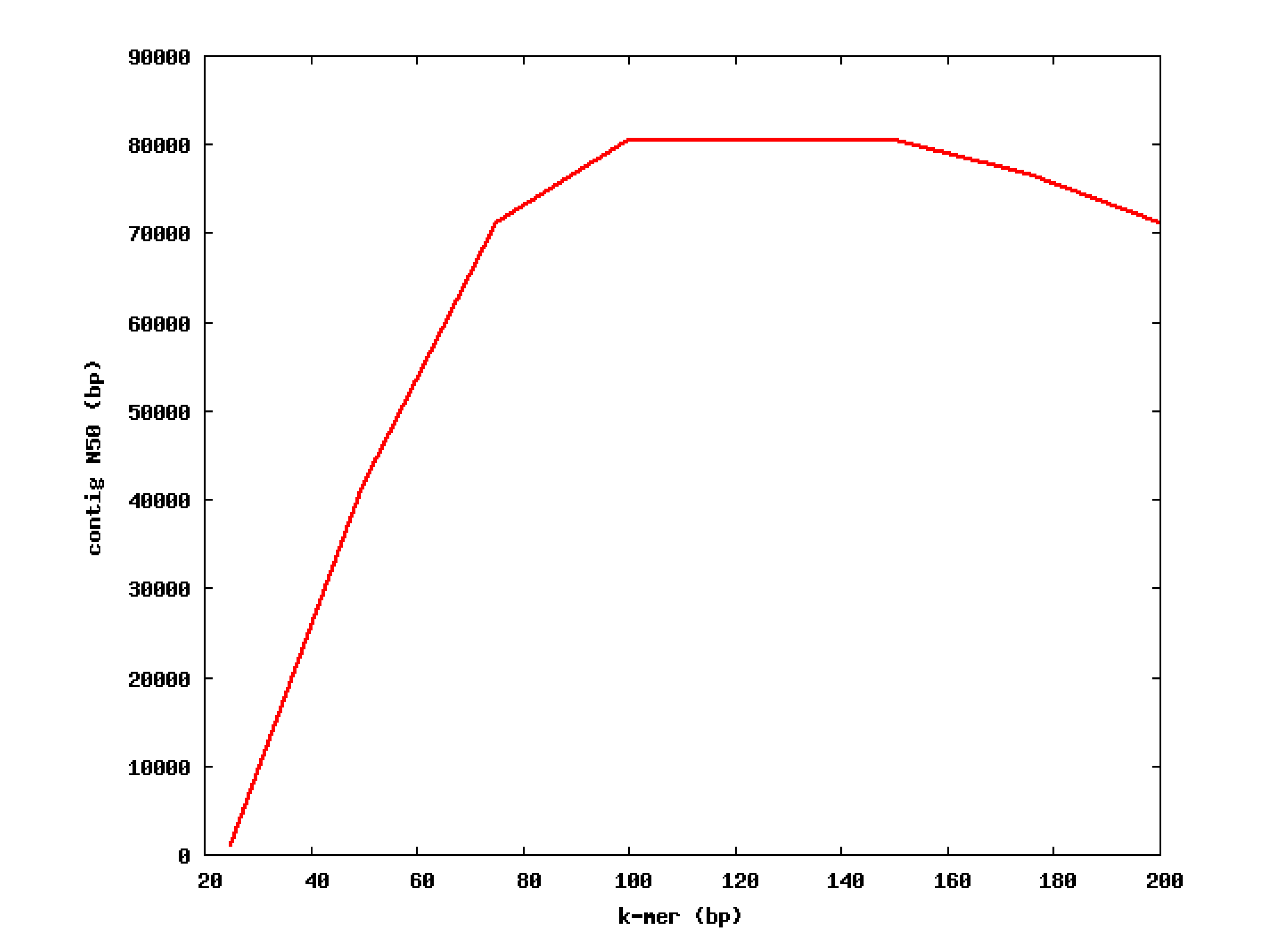

Supplement: Figure S1 — Relationship between contig N50 length and data coverage. The results are for the SOAPdenovo assemblies (with a k-mer value 51) on the original PE data of P. brasiliensis. (TIFF) [file pone.0069503.s001.tiff]

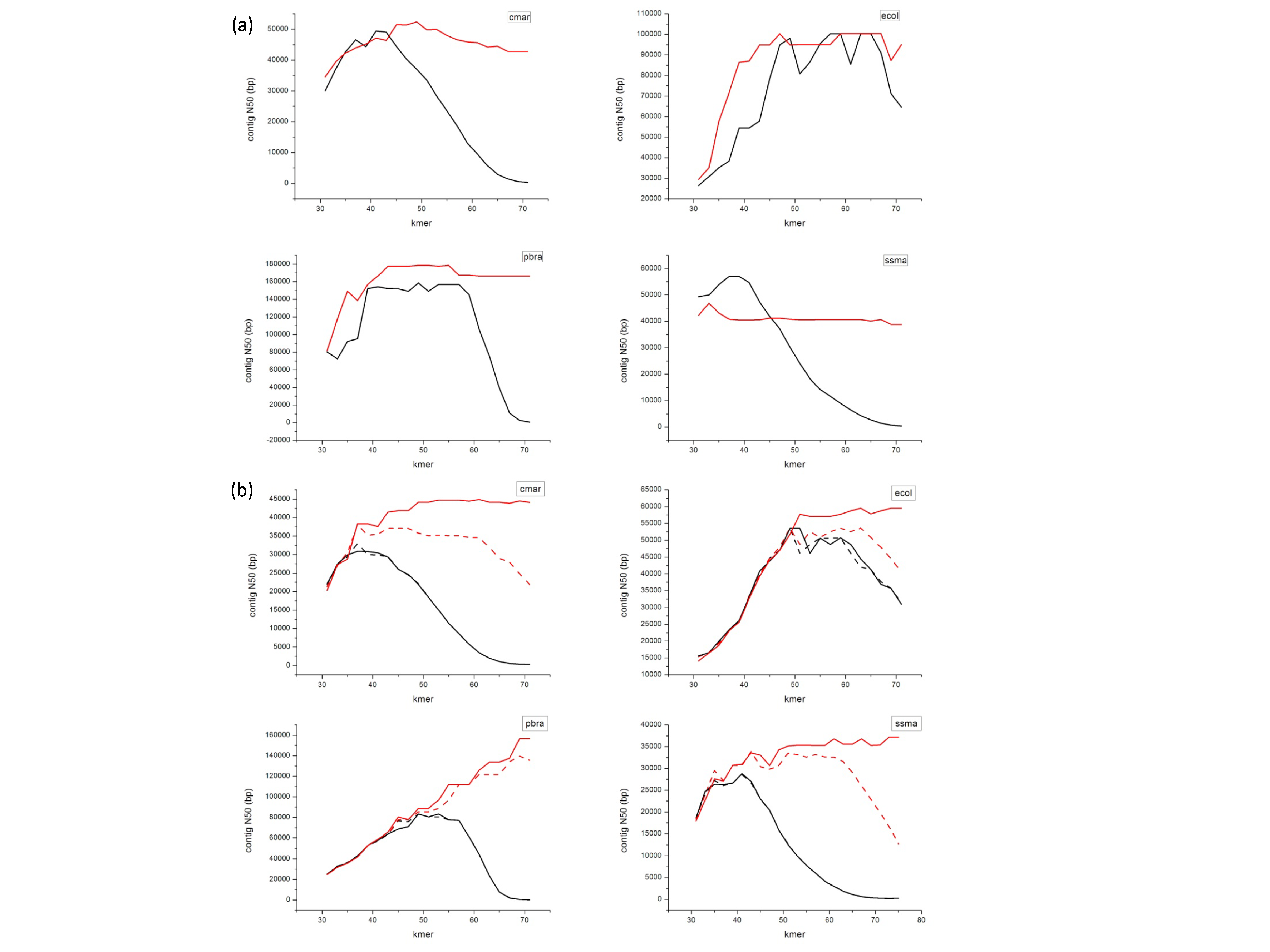

Supplement: Figure S2 — Relationship between contig N50 length and k-mer value. For (a) Velvet and (b) SOAPdenovo, we scan various k-mer values to optimize four bacterial assemblies. The black and red curves represent the assembly of original PEs and with recovered DNA fragments, respectively. For SOAPdenovo, the solid and dashed lines are for the assemblies where the parameter maximal read lengths are set and not, respectively. (TIFF) [file pone.0069503.s002.tiff]

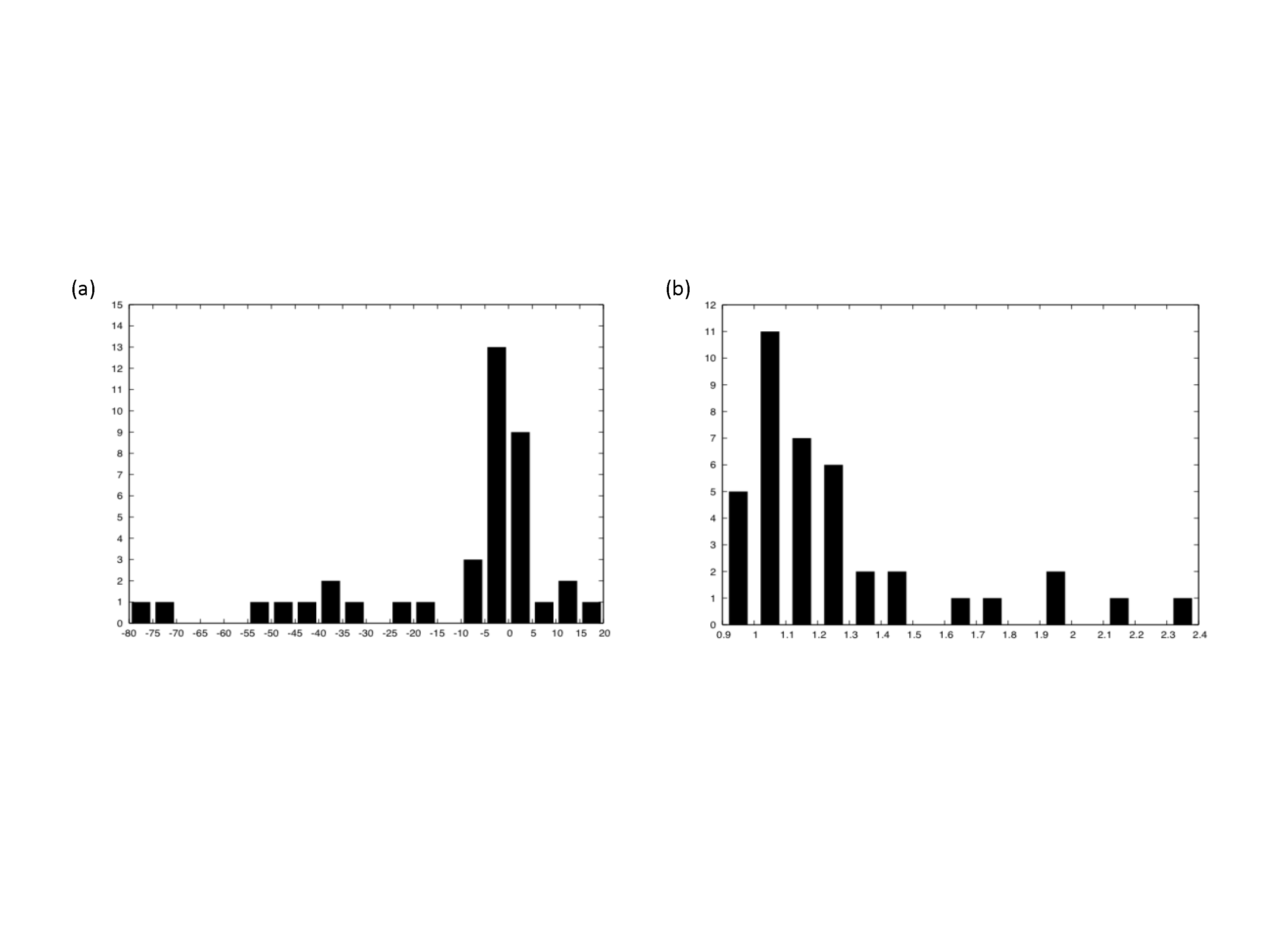

Supplement: Figure S3 — Effects of error correction and read filtering on assembly. (a) Histogram of differences in number of errors after error correction and read filtering by ARF-PE on the real data of the four bacteria. A negative number stands for a reduction in number of errors. (b) Histogram of the ratio in corrected N50 length. A ratio greater than one means an increase in corrected N50 length after applying the two options of ARF-PE. Note that on real data, the CABOG assembly of recovered DNA fragments and the original PEs of E. coli aborted without a clear reason, so there are 39 cases in total. (TIF) [file pone.0069503.s003.tif]

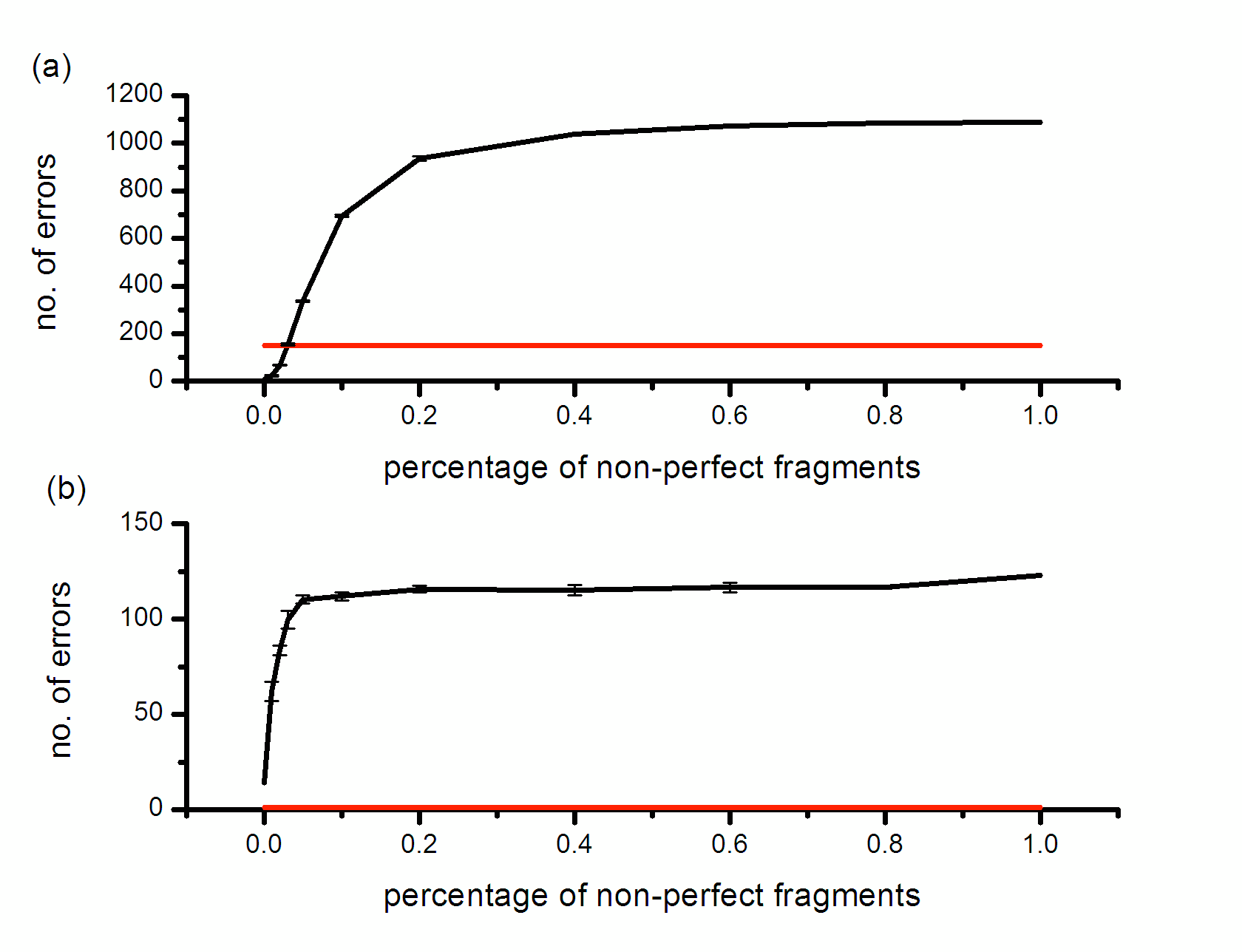

Supplement: Figure S4 — Assembly errors by various fractions of non-perfectly recovered DNA fragments. For (a) N. crassa and (b) human chromosome 22, we separate the perfectly recovered DNA fragments from the non-perfect ones. Different fractions of non-perfect fragments (x-axis) and all the perfect ones are assembled by SOAPdenovo (with kmer = 127), and the number of errors (y-axis) are computed by the GAGE script. For each fraction, reads are randomly selected three times for assembly; the mean and variation in number of errors are shown. Horizontal red lines indicate the number of errors when the original PEs are assembled. (TIF) [file pone.0069503.s004.tif]
