# Supplementary material for: Optimizing Information in Next-Generation-Sequencing (NGS) Reads for Improving De Novo Genome Assembly
Source: PLoS One. 2013 Jul 29;8(7):e69503. doi: 10.1371/journal.pone.0069503 (PMC3726674; doi:10.1371/journal.pone.0069503)
Supplement: Table S1 — Performance of ARF-PE on the simulated PE libraries of four bacteria. The four species are (a) C. marinum, (b) E. coli, (c), P. brasiliensis, and (d) S. smaragdinae. The definitions of percentages follow those in Table 4. (DOCX) [file pone.0069503.s005.docx]

| (a) | | | | | |
| --- | --- | --- | --- | --- | --- |
| PE read mappings | Regular | Bridging | Single-mappable-end | Unmapped | Total |
| No. (%) of PEs | 3,972,890 (97.07%) | 24,331 (0.59%) | 93,477 (2.28%) | 2,245 (0.05%) | 4,092,943 (100%) |
| No. (%) of recovered fragments | 3,972,890 (100.00%) | 17,814 (73.22%) | 90,138 (96.43%) | N.A. | 4,080,842 (99.70%) |
| No. (%) of correctly recovered fragments | 3,970,478 (99.94%) | 16,945 (95.12%) | 84,550 (93.80%) | N.A. | 4,071,973 (99.78%) |
| No. (%) of perfectly recovered fragments | 3,948,863 (99.40%) | 12,772 (71.70%) | 78,467 (87.05%) | N.A. | 4,040,102 (99.00%) |
| (b) | | | | | |
| No. (%) of PEs | 2,214,677 (95.47%) | 32,804 (1.41%) | 65,033 (2.80%) | 7,324 (0.32%) | 2,319,838 (100%) |
| No. (%) of recovered fragments | 2,214,677 (100.00%) | 28,015 (85.40%) | 48,695 (74.88%) | N.A. | 2,291,387 (98.77%) |
| No. (%) of correctly recovered fragments | 2,211,883 (99.87%) | 27,015 (96.43%) | 45,632 (93.71%) | N.A. | 2,284,530 (99.70%) |
| No. (%) of perfectly recovered fragments | 2,203,123 (99.48%) | 19,295 (68.87%) | 39,041 (80.17%) | N.A. | 2,261,459 (98.69%) |
| (c) | | | | | |
| No. (%) of PEs | 3,844,542 (97.29%) | 21,131 (0.53%) | 83,820 (2.12%) | 2219 (0.06%) | 3,951,712 (100%) |
| No. (%) of recovered fragments | 3,844,542 (100.00%) | 16,436 (77.78%) | 80,777 (96.37%) | N.A. | 3,941,755 (99.75%) |
| No. (%) of correctly recovered fragments | 3,842,518 (99.95%) | 16,302 (99.18%) | 77,930 (96.48%) | N.A. | 3,936,750 (99.87%) |
| No. (%) of perfectly recovered fragments | 3,828,589 (99.59%) | 11,875 (72.25%) | 72,295 (89.50%) | N.A. | 3,912,759 (99.26%) |
| (d) | | | | | |
| No. (%) of PEs | 2,925,760 (95.56%) | 19,754 (0.65%) | 104,554 (3.41%) | 11,755 (0.38%) | 3,061,823 (100%) |
| No. (%) of recovered fragments | 2,925,760 (100.00%) | 15,123 (76.56%) | 86,923 (83.14%) | N.A. | 3,027,806 (98.89%) |
| No. (%) of correctly recovered fragments | 2,923,865 (99.94%) | 14,395 (95.19%) | 83,624 (96.20%) | N.A. | 3,021,884 (99.80%) |
| No. (%) of perfectly recovered fragments | 2,907,949 (99.39%) | 8,880 (58.72%) | 73,225 (84.24%) | N.A. | 2,990,054 (98.75%) |
