# Supplementary material for: Optimizing Information in Next-Generation-Sequencing (NGS) Reads for Improving De Novo Genome Assembly
Source: PLoS One. 2013 Jul 29;8(7):e69503. doi: 10.1371/journal.pone.0069503 (PMC3726674; doi:10.1371/journal.pone.0069503)
Supplement: Table S2 — Assembly statistics on the simulated data of four bacteria by three assemblers. We use SOAPdenovo, SOAPdenovo+GapCloser, and Newbler to assemble three types of simulated data of four bacteria: (a) C. marinum, (b) E. coli, (c), P. brasiliensis, and (d) S. smaragdinae. The three types of data are (1) original PE reads, (2) recovered DNA fragments and the remaining PEs, and (3) recovered DNA fragments and original PE reads. (DOCX) [file pone.0069503.s006.docx]

| (a) | | | | | | | |
| --- | --- | --- | --- | --- | --- | --- | --- |
| Assembler | Data | Total contig length (bp) | No. of contigs | N50 (bp) | No. of errors | N50 corr. (bp) | Accuracy (%) |
| SOAPdenovo | original PEs | 6194936 | 263 | 104832 | 0 | 104832 | 100.00 |
|  | recovered fragments + remaining PEs | 6191559 | 204 | 155574 | 13 | 149856 | 96.32 |
|  | recovered fragments + original PEs | 6191559 | 204 | 155574 | 13 | 149856 | 96.32 |
| SOAPdenovo + GapCloser | original PEs | 6171773 | 97 | 299345 | 3 | 185996 | 62.13 |
|  | recovered fragments + remaining PEs | 6174960 | 209 | 137814 | 10 | 137814 | 100.00 |
|  | recovered fragments + original PEs | 6178915 | 109 | 231275 | 2 | 172365 | 74.53 |
| Newbler | original PEs | 6118048 | 300 | 67027 | 54 | 53363 | 79.61 |
|  | recovered fragments + remaining PEs | 6151403 | 141 | 171150 | 7 | 171150 | 100.00 |
|  | recovered fragments + original PEs | 6145761 | 145 | 171075 | 3 | 171075 | 100.00 |
| (b) | | | | | | | |
| SOAPdenovo | original PEs  recovered fragments + remaining PEs  recovered fragments + original PEs | 4590559  4550412  4551490 | 256  165  167 | 57832  110962  110962 | 0  23  23 | 57832  73906  73906 | 100.00  66.60  66.60 |
| SOAPdenovo + GapCloser | original PEs  recovered fragments + remaining PEs  recovered fragments + original PEs | 4553424  4548688  4547651 | 104  155  104 | 132904  112427  133403 | 17  23  15 | 105923  82832  95420 | 79.70  73.68  71.53 |
| Newbler | original PEs  recovered fragments + remaining PEs  recovered fragments + original PEs | 4544162  4555170  4553744 | 179  112  160 | 62107  125646  95343 | 10  13  7 | 58665  105598  78499 | 94.46  84.04  82.33 |
| (c) | | | | | | | |
| SOAPdenovo | original PEs  recovered fragments + remaining PEs  recovered fragments + original PEs | 5985166  5985261  6191559 | 214  146  204 | 124055  193636  162063 | 0  4  0 | 124055  156863  162063 | 100.00  81.01  100.00 |
| SOAPdenovo + GapCloser | original PEs  recovered fragments + remaining PEs  recovered fragments + original PEs | 5969274  5976378  6178915 | 77  157  109 | 218604  166489  231275 | 6  7  0 | 190980  156606  231275 | 87.36  94.06  100.00 |
| Newbler | original PEs  recovered fragments + remaining PEs  recovered fragments + original PEs | 5931886  5956969  5953302 | 299  102  104 | 45745  193388  165897 | 40  3  4 | 40444  166027  165897 | 88.41  85.85  100.00 |
| (d) | | | | | | | |
| SOAPdenovo | original PEs  recovered fragments + remaining PEs  recovered fragments + original PEs | 4645059  4604065  4604065 | 332  189  189 | 97328  162904  162904 | 0  9  9 | 97328  162904  162904 | 100.00  100.00  100.00 |
| SOAPdenovo + GapCloser | original PEs  recovered fragments + remaining PEs  recovered fragments + original PEs | 4615728  4615728  4615728 | 114  199  117 | 194902  138382  195075 | 2  2  2 | 194902  N.A.  194902 | 100.00  N.A.  99.91 |
| Newbler | original PEs  recovered fragments + remaining PEs  recovered fragments + original PEs | 4638865  4612676  4610333 | 247  154  167 | 65080  186268  183745 | 52  3  8 | 46381  186268  183745 | 71.27  100.00  100.00 |
