# Supplementary material for: Optimizing Information in Next-Generation-Sequencing (NGS) Reads for Improving De Novo Genome Assembly
Source: PLoS One. 2013 Jul 29;8(7):e69503. doi: 10.1371/journal.pone.0069503 (PMC3726674; doi:10.1371/journal.pone.0069503)
Supplement: Table S3 — ARF-PE runtime on the PE libraries in this study. The run times are in wall clock seconds. The data of the four bacteria and S. cerevisiae are run on a server with four Quad-Core Intel Xeon CPUs E5520 (2.26 GHz) and one with two Quad-Core AMD Opteron processors 2378 (800 MHz), respectively. The N. crassa and human data are run on a server with eight Oct-Core Intel Xeon CPUs E7-4820 (2.00 GHz). Note that for each dataset, the total amount of time is not equal to the sum of the section times because ARF-PE spends extra time for data transformation and re-collection. (DOCX) [file pone.0069503.s007.docx]

| Species | Read filtering | Initial assembly | Error correction | Kernel | Total |
| --- | --- | --- | --- | --- | --- |
| *C. marinum* | 308 | 428 | 1047 | 3497 | 5312 |
| *E. coli* | 226 | 304 | 734 | 2243 | 3528 |
| *P. brasiliens* | 295 | 412 | 983 | 4226 | 5946 |
| *S. smaragdinae* | 232 | 312 | 809 | 3143 | 4521 |
| *S. cerevisiae* | 228 | 245 | 1064 | 4905 | 6463 |
| *N. crassa* | 3998 | 8787 | 10800 | 97600 | 122062 |
| Human chr22 | N.A. | 2417 | 4887 | 36694 | 44249 |
