# Supplementary material for: Optimizing Information in Next-Generation-Sequencing (NGS) Reads for Improving De Novo Genome Assembly
Source: PLoS One. 2013 Jul 29;8(7):e69503. doi: 10.1371/journal.pone.0069503 (PMC3726674; doi:10.1371/journal.pone.0069503)
Supplement: Table S4 — Performance of ARF-PE on the real PE libraries of four bacteria. The four species are (a) C. marinum, (b) E. coli, (c), P. brasiliensis, and (d) S. smaragdinae. (DOCX) [file pone.0069503.s008.docx]

| (a) | | | | | |
| --- | --- | --- | --- | --- | --- |
| PE read mappings | Regular | Bridging | Single-mappable-end | Unmapped | Total |
| No. (%) of PEs | 3697276 (90.35%) | 24275 (0.59%) | 313873 (7.67%) | 56822 (1.39%) | 4092246 (100%) |
| No. (%) of recovered fragments | 3697276 (100.00%) | 9528 (39.25%) | 176334 (56.18%) | N.A. | 3883138 (94.89%) |
| No. (%) of correctly recovered fragments | 3694186 (99.92%) | 9320 (97.82%) | 173942 (98.64%) | N.A. | 3877448 (99.85%) |
| No. (%) of perfectly recovered fragments | 3691297 (99.84%) | 7504 (78.76%) | 168465 (95.54%) | N.A. | 3867266 (99.59%) |
| (b) | | | | | |
| No. (%) of PEs | 2181071 (94.04%) | 37679 (1.62%) | 81922 (3.53%) | 18656 (0.80%) | 2319328 (100%) |
| No. (%) of recovered fragments | 2181071 (100.00%) | 28368 (75.29%) | 43147 (52.67%) | N.A. | 2252586 (97.12%) |
| No. (%) of correctly recovered fragments | 2176753 (99.80%) | 27102 (95.54%) | 40818 (94.60%) | N.A. | 2244673 (99.65%) |
| No. (%) of perfectly recovered fragments | 2173053 (99.63%) | 16613 (58.56%) | 35720 (82.79%) | N.A. | 2225386 (98.79%) |
| (c) | | | | | |
| No. (%) of PEs | 3319754 (84.13%) | 178443 (4.52%) | 245865 (6.23%) | 201715 (5.11%) | 3945777 (100%) |
| No. (%) of recovered fragments | 3319754 (100.00%) | 9969 (5.59%) | 113629 (46.22%) | N.A. | 3443352 (87.27%) |
| No. (%) of correctly recovered fragments | 3318586 (99.96%) | 9949 (99.80%) | 113032 (99.47%) | N.A. | 3441567 (99.95%) |
| No. (%) of perfectly recovered fragments | 3316999 (99.92%) | 8194 (82.19%) | 111078 (97.75%) | N.A. | 3436271 (99.79%) |
| (d) | | | | | |
| No. (%) of PEs | 2717383 (88.76%) | 18959 (0.62%) | 283761 (9.27%) | 41436 (1.35%) | 3061539 (100%) |
| No. (%) of recovered fragments | 2717383 (100.00%) | 11471 (60.50%) | 99422 (35.04%) | N.A. | 2828276 (92.38%) |
| No. (%) of correctly recovered fragments | 2712448 (99.82%) | 11301 (98.52%) | 98233 (98.80%) | N.A. | 2821982 (99.78%) |
| No. (%) of perfectly recovered fragments | 2709661 (99.72%) | 7787 (67.88%) | 90342 (90.87%) | N.A. | 2807790 (99.28%) |
