# Supplementary material for: Optimizing Information in Next-Generation-Sequencing (NGS) Reads for Improving De Novo Genome Assembly
Source: PLoS One. 2013 Jul 29;8(7):e69503. doi: 10.1371/journal.pone.0069503 (PMC3726674; doi:10.1371/journal.pone.0069503)
Supplement: Table S5 — Assembly statistics on the real data of four bacteria. We use Velvet, SOAPdenovo, SOAPdenovo+GapCloser, Newbler, and CABOG for assembling three types of real data of four bacteria: (a) C. marinum, (b) E. coli, (c), P. brasiliensis, and (d) S. smaragdinae. (DOCX) [file pone.0069503.s009.docx]

| (a) | | | | | | | |
| --- | --- | --- | --- | --- | --- | --- | --- |
| Assembler | Data | Total contig length (bp) | No. of contigs | N50 (bp) | No. of errors | N50 corr. (bp) | Accuracy (%) |
| Velvet | original PEs | 6145624 | 296 | 47761 | 6 | 46585 | 97.54 |
|  | recovered fragments + remaining PEs | 6143481 | 308 | 50422 | 18 | 46555 | 92.33 |
|  | recovered fragments + original PEs | 6141422 | 264 | 52271 | 15 | 49937 | 95.53 |
| SOAPdenovo | original PEs | 6223447 | 532 | 30840 | 1 | 30076 | 97.52 |
|  | recovered fragments + remaining PEs | 6180602 | 380 | 44695 | 10 | 44116 | 98.70 |
|  | recovered fragments + original PEs | 6201628 | 386 | 44690 | 3 | 44443 | 99.45 |
| SOAPdenovo + GapCloser | original PEs | 6165344 | 206 | 129179 | 27 | 100589 | 77.87 |
|  | recovered fragments + remaining PEs | 6163862 | 266 | 75273 | 18 | 65232 | 86.66 |
|  | recovered fragments + original PEs | 6165769 | 191 | 102485 | 26 | 83177 | 81.16 |
| Newbler | original PEs | 6118679 | 362 | 42700 | 77 | 33414 | 78.25 |
|  | recovered fragments + remaining PEs | 6144214 | 209 | 97774 | 7 | 80984 | 82.83 |
|  | recovered fragments + original PEs | 6140524 | 210 | 96608 | 7 | 80941 | 83.78 |
| CABOG | original PEs | 6142896 | 213 | 50500 | 42 | 43797 | 86.73 |
|  | recovered fragments + remaining PEs | 6162069 | 93 | 151340 | 33 | 102357 | 67.63 |
|  | recovered fragments + original PEs | 6159456 | 77 | 207198 | 34 | 147569 | 71.22 |
| (b) | | | | | | | |
| Velvet | original PEs  recovered fragments + remaining PEs  recovered fragments + original PEs | 4542997  4534349  4534088 | 135  138  147 | 95343  100392  100403 | 10  21  21 | 71861  65836  66333 | 75.37  65.58  66.07 |
| SOAPdenovo | original PEs  recovered fragments + remaining PEs  recovered fragments + original PEs | 4591824  4567726  4568135 | 278  194  194 | 52516  85529  85531 | 2  18  18 | 52516  65836  65836 | 100.00  76.98  76.97 |
| SOAPdenovo + GapCloser | original PEs  recovered fragments + remaining PEs  recovered fragments + original PEs | 4572601  4564680  4581118 | 139  172  139 | 111835  101340  110246 | 9  20  11 | 88292  80652  100313 | 78.95  79.59  90.99 |
| Newbler | original PEs  recovered fragments + remaining PEs  recovered fragments + original PEs | 4539050  4553244  4554689 | 160  114  147 | 80377  123795  88995 | 16  17  11 | 70922  95347  82669 | 88.24  77.02  92.89 |
| CABOG | original PEs  recovered fragments + remaining PEs  recovered fragments + original PEs | 4574034  4607596  4675982 | 149  84  407 | 64448  126692  26864 | 689  32  26 | 9415  74772  24234 | 14.61  59.02  90.21 |
| (c) | | | | | | | |
| Velvet | original PEs  recovered fragments + remaining PEs  recovered fragments + original PEs | 5966809  5965863  5966059 | 108  131  97 | 158581  189520  178598 | 6  10  8 | 121638  156596  166495 | 76.70  82.63  93.22 |
| SOAPdenovo | original PEs  recovered fragments + remaining PEs  recovered fragments + original PEs | 5989326  5982458  5982458 | 232  135  135 | 80524  166573  166573 | 1  5  5 | 76637  140796  140796 | 95.17  84.53  84.53 |
| SOAPdenovo + GapCloser | original PEs  recovered fragments + remaining PEs  recovered fragments + original PEs | 5976599  5972491  5971531 | 144  139  102 | 190328  178240  178469 | 8  7  9 | 152395  156594  166489 | 80.07  87.86  93.29 |
| Newbler | original PEs  recovered fragments + remaining PEs  recovered fragments + original PEs | 5941846  5957736  5955141 | 189  93  96 | 80655  193388  193388 | 19  6  6 | 64086  156799  190871 | 79.46  81.08  98.70 |
| CABOG | original PEs  recovered fragments + remaining PEs  recovered fragments + original PEs | 5958614  5975578  5979373 | 71  49  45 | 204964  280361  281920 | 23  13  15 | 140673  177912  178146 | 68.63  63.46  63.19 |
| (d) | | | | | | | |
| Velvet | original PEs  recovered fragments + remaining PEs  recovered fragments + original PEs | 4570455  4542097  4541633 | 255  317  296 | 56427  41808  45916 | 5  6  8 | 56007  41808  45448 | 99.26  100.00  98.98 |
| SOAPdenovo | original PEs  recovered fragments + remaining PEs  recovered fragments + original PEs | 4664048  4664682  4596902 | 499  380  340 | 28998  41353  37214 | 2  1  4 | 28998  41342  37214 | 100.00  99.97  100.00 |
| SOAPdenovo + GapCloser | original PEs  recovered fragments + remaining PEs  recovered fragments + original PEs | 4621131  4610662  4616708 | 189  257  157 | 121053  100304  162756 | 6  3  3 | 121053  100304  162756 | 100.00  100.00  100.00 |
| Newbler | original PEs  recovered fragments + remaining PEs  recovered fragments + original PEs | 4588100  4610083  4607870 | 411  225  215 | 29296  60871  77713 | 62  9  4 | 24064  60515  77713 | 82.14  99.42  100.00 |
| CABOG | original PEs  recovered fragments + remaining PEs  recovered fragments + original PEs | 4166068  4119026  4094712 | 179  71  66 | 41962  104981  105056 | 32  26  26 | 36100  86454  86454 | 86.03  82.35  82.29 |
