# Supplementary material for: Optimizing Information in Next-Generation-Sequencing (NGS) Reads for Improving De Novo Genome Assembly
Source: PLoS One. 2013 Jul 29;8(7):e69503. doi: 10.1371/journal.pone.0069503 (PMC3726674; doi:10.1371/journal.pone.0069503)
Supplement: Table S6 — Assembly statistics on the real data of two fungi. The two fungi are (a) S. cerevisiae, and (b) N. crassa. (DOCX) [file pone.0069503.s010.docx]

| (a) | | | | | | | |
| --- | --- | --- | --- | --- | --- | --- | --- |
| Assembler | Data | Total contig length (bp) | No. of contigs | N50 (bp) | No. of errors | N50 corr. (bp) | Accuracy (%) |
| SOAPdenovo | original PEs | 11660407 | 2282 | 10871 | 11 | 10860 | 99.90 |
|  | recovered fragments + remaining PEs | 11683977 | 2075 | 12416 | 13 | 12343 | 99.41 |
|  | recovered fragments + original PEs | 11725403 | 2006 | 12973 | 13 | 12901 | 99.45 |
| SOAPdenovo + GapCloser | original PEs | 11696423 | 1361 | 21506 | 47 | 20463 | 95.15 |
|  | recovered fragments + remaining PEs | 11681637 | 1804 | 16144 | 36 | 15623 | 96.77 |
|  | recovered fragments + original PEs | 11700539 | 1298 | 22724 | 43 | 21354 | 93.97 |
| Newbler | recovered fragments | 11496825 | 1388 | 16240 | 35 | 15693 | 96.63 |
|  | recovered fragments + remaining PEs | 11696153 | 1049 | 30652 | 30 | 29470 | 96.14 |
|  | recovered fragments + original PEs | 11695170 | 1059 | 30652 | 32 | 29470 | 96.14 |
| (b) | | | | | | | |
| SOAPdenovo | original PEs  recovered fragments + remaining PEs  recovered fragments + original PEs | 37837899  34462815  34462815 | 19879  7923  7923 | 3420  7233  7233 | 150  1095  1095 | 3388  6604  6604 | 99.06  91.30  91.30 |
| SOAPdenovo + GapCloser | original PEs  recovered fragments + remaining PEs | 38917477  34679314 | 8034  6358 | 15332  10326 | 1477  1517 | 11072  8128 | 72.21  78.71 |
|  | recovered fragments + original PEs | 38954433 | 8061 | 17384 | 2241 | 10574 | 60.83 |
| Newbler | recovered fragments | 34515973 | 8108 | 7291 | 1125 | 6656 | 91.29 |
