# Supplementary material for: Optimizing Information in Next-Generation-Sequencing (NGS) Reads for Improving De Novo Genome Assembly
Source: PLoS One. 2013 Jul 29;8(7):e69503. doi: 10.1371/journal.pone.0069503 (PMC3726674; doi:10.1371/journal.pone.0069503)
Supplement: Table S7 — Assembly statistics on the simulated data of human chromosome 22. (DOCX) [file pone.0069503.s011.docx]

| Assembler | Data | Total contig length (bp) | No. of contigs | N50 (bp) | No. of errors | N50 corr. (bp) | Accuracy (%) |
| --- | --- | --- | --- | --- | --- | --- | --- |
| SOAPdenovo | original PEs | 35658342 | 7635 | 15534 | 1 | 15523 | 99.93 |
|  | recovered fragments + remaining PEs | 34660466 | 3685 | 50886 | 123 | 46785 | 91.94 |
|  | recovered fragments + original PEs | 34660466 | 3685 | 50886 | 123 | 46785 | 91.94 |
| SOAPdenovo + GapCloser | original PEs | 34861844 | 1627 | 526386 | 209 | 176720 | 33.57 |
|  | recovered fragments + remaining PEs | 34686875 | 3172 | 78812 | 150 | 64220 | 81.49 |
|  | recovered fragments + original PEs | 34923156 | 1648 | 542034 | 205 | 200383 | 36.97 |
| Newbler | recovered fragments + remaining PEs | 34085423 | 1687 | 76792 | 132 | 62597 | 81.52 |
|  | 20% recovered fragments + remaining PEs : 1 | 34074424 | 1643 | 77012 | 124 | 65882 | 85.55 |
|  | 20% recovered fragments + remaining PEs : 2 | 34069010 | 1643 | 70274 | 133 | 62602 | 89.08 |
|  | 20% recovered fragments + remaining PEs : 3 | 34059587 | 1634 | 71508 | 127 | 64226 | 89.82 |
